# Photosynthesis in *Synechocystis* sp. PCC 6803 is not optimally regulated under very high CO_2_

**DOI:** 10.1007/s00253-025-13416-2

**Published:** 2025-01-30

**Authors:** Elena Carrasquer-Alvarez, Ute Angelika Hoffmann, Adrian Sven Geissler, Axel Knave, Jan Gorodkin, Stefan Ernst Seemann, Elton P. Hudson, Niels-Ulrik Frigaard

**Affiliations:** 1 Marine Biological Section, Department of Biology, University of Copenhagen, Helsingør, Denmark; 2 School of Engineering Sciences in Chemistry, Biotechnology and Health, Science for Life Laboratory, KTH—Royal Institute of Technology, Stockholm, Sweden; 3 Center for Non-Coding RNA in Technology and Health, Department of Veterinary and Animal Sciences, University of Copenhagen, Frederiksberg, Denmark

**Keywords:** CRISPR technology, Carbon sequestration, Stress tolerance, High CO_2_ concentrations, Metabolic engineering, Photosynthesis regulation

## Abstract

**Abstract:**

One strategy for CO_2_ mitigation is using photosynthetic microorganisms to sequester CO_2_ under high concentrations, such as in flue gases. While elevated CO_2_ levels generally promote growth, excessively high levels inhibit growth through uncertain mechanisms. This study investigated the physiology of the cyanobacterium *Synechocystis* sp. PCC 6803 under very high CO_2_ concentrations and yet stable pH around 7.5. The growth rate of the wild type (WT) at 200 µmol photons m^−2^ s^−1^ and a gas phase containing 30% CO_2_ was 2.7-fold lower compared to 4% CO_2_. Using a CRISPR interference mutant library, we identified genes that, when repressed, either enhanced or impaired growth under 30% or 4% CO_2_. Repression of genes involved in light harvesting (*cpc* and *apc*), photochemical electron transfer (*cytM*, *psbJ*, and *petE*), and several genes with little or unknown functions promoted growth under 30% CO_2_, while repression of key regulators of photosynthesis (*pmgA*) and CO_2_ capture and fixation (*ccmR*, *cp12*, and *yfr1*) increased growth inhibition under 30% CO_2_. Experiments confirmed that WT cells were more susceptible to light inhibition under 30% than under 4% CO_2_ and that a light-harvesting-impaired Δ*cpcG* mutant showed improved growth under 30% CO_2_ compared to the WT. These findings suggest that enhanced fitness under very high CO_2_ involves modifications in light harvesting, electron transfer, and carbon metabolism, and that the native regulatory machinery is insufficient, and in some cases obstructive, for optimal growth under 30% CO_2_. This genetic profiling provides potential targets for engineering cyanobacteria with improved photosynthetic efficiency and stress resilience for biotechnological applications.

**Key points:**

• *Synechocystis growth was inhibited under very high CO*_*2*_*.*

• *Inhibition of growth under very high CO*_*2*_* was light dependent.*

• *Repression of photosynthesis genes improved growth under very high CO*_*2*_*.*

**Graphical Abstract:**

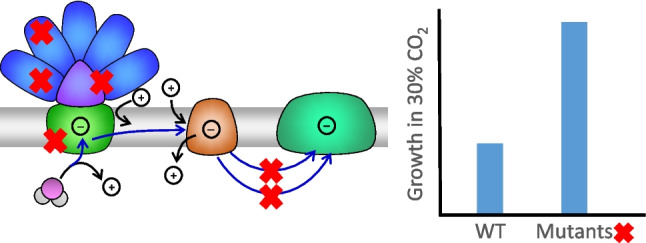

**Supplementary Information:**

The online version contains supplementary material available at 10.1007/s00253-025-13416-2.

## Introduction

Cyanobacteria and microalgae are the only microbes with a plant-like and oxygenic photosynthesis and are responsible for roughly half of the CO_2_ that is converted to biomass on Earth (Kirchman 2018). These organisms also constitute promising platforms for sustainable, biotechnological production of food, feed, biofuel, and high-value bioproducts such as pigments, antioxidants, and bioactive compounds (Zhou et al. 2017; Klemenčič et al. 2017). In nature, they primarily use CO_2_ as the sole carbon source for growth and sunlight as the sole source of energy (Kirchman 2018). In their natural environments, growth easily becomes limited by CO_2_ because the CO_2_ content in air (0.04%) is relatively low, and the diffusion of CO_2_ from the gas phase to cells in the liquid phase is slow. Therefore, for optimal cultivation of these microbes, CO_2_ is often added to the gas phase of the culture in concentrations of 1–5% CO_2_ (Jaiswal and Kashyap 2002; Zavřel et al. 2017; Zhou et al. 2017). Unfortunately, concentrations of CO_2_ higher than roughly 5% in the gas phase may cause inhibition of the microbes, even if the pH is kept around the optimal, which typically is around pH 7–8 (Zhou et al. 2017; Zhao and Su 2014; de Morais and Costa 2007; Jaiswal and Kashyap 2002; Stöckel et al. 2013). The CO_2_ concentrations that inhibit specific cyanobacterial and microalgal strains vary widely for reasons that are not clear (Zhou et al. 2017; Zhao and Su 2014). The CO_2_ that is supplied to cultures of photosynthetic microbes must, therefore, be appropriately diluted, typically by air, to avoid growth inhibition by CO_2_. Nevertheless, there may be several advantages if high CO_2_ concentrations could be used for cultivation purposes. For example, (1) gases with high CO_2_ concentrations, such as flue gas (roughly 15% CO_2_) and fermentation exhaust (up to nearly 100% CO_2_) could be used with less dilution; (2) production of certain desirable bioproducts may be enhanced in very high CO_2_ conditions; (3) photorespiration (which limits net photosynthetic productivity) may be suppressed; and (4) high CO_2_ concentrations may inhibit undesirable microbes in the culture (photosynthetic as well as non-photosynthetic). Therefore, it could be beneficial for practical applications of photosynthetic microbes if the strains of interest were tolerant to very high CO_2_ concentrations. In this work, we investigated physiological explanations for the inhibition of cyanobacteria by very high CO_2_ and biological strategies to overcome this inhibition. This knowledge will not only increase our understanding of microbial CO_2_ metabolism but also help in the identification of genetic and physiological mechanisms that can enhance the productivity and resilience of these microbes for industrial and biotechnological applications.

While many studies have investigated the physiological adaptations of cyanobacteria under conditions of limiting and optimal CO_2_ concentrations (e.g., Ludwig and Bryant 2012; Lieman-Hurwitz et al. 2009; Knoop et al. 2010; Spät et al. 2021), little attention has been given to the effects of inhibitory high CO_2_ concentrations. In several studies testing very high CO_2_ concentrations, the pH has not been controlled, resulting in growth-inhibitory acidification of the cultivation medium (e.g., Hanagata et al. 1992; Jaiswal and Kashyap 2002; Yue and Chen 2005; Ota et al. 2009). Hence, in these studies, it may be difficult to distinguish the inhibitory effects of low pH from those of high CO_2_. In addition, in experimental setups examining the effect of high CO_2_ contents in the gas phase on a culture in the liquid phase, it is important to use sufficient mixing between the gas and liquid phases (typically through vigorous bubbling) to ensure a stable and predictable CO_2_ concentration in the liquid medium (Zheng et al. 2018). Only a few studies have examined the physiological effects of inhibitory high CO_2_ concentrations on cyanobacteria. For example, Stöckel et al. (2013) analyzed the transcriptomic responses in the marine, nitrogen-fixing *Cyanothece* sp. ATCC 51142 under light/dark cycling and limiting CO_2_ (air; 0.04%) and growth-inhibitory CO_2_ (8%) concentrations (the transcriptome under optimal growth conditions at 1% CO_2_ was not investigated). They found that, in the light, genes associated with photosynthesis (antenna and electron transfer) and CO_2_ concentrating mechanisms exhibited reduced transcript levels under 8% CO_2_ when compared to air. In contrast, (also in the light) genes related to respiration, synthesis of carbon-rich compounds (extracellular polysaccharides and glycogen), and degradation of phycobilisomes (PBS) were upregulated in 8% CO_2_ conditions when compared to air. These responses in *Cyanothece* were partially attributed to an increased biomass C/N ratio under 8% CO_2_ conditions compared to air. In another study, Mehta et al. (2019) investigated the proteome of the freshwater *Synechococcus elongatus* PCC 11801 under optimal (0.5%) and growth-inhibitory (10%) CO_2_ concentrations (without pH control of the medium). They found that photosynthesis (represented by the protein components of PBS, photosystems (PS) I and II, electron transfer, CO_2_ fixation, and chlorophyll biosynthesis) was downregulated in 10% CO_2_. A few proteins were upregulated in 10% CO_2_ compared to optimal conditions, including superoxide dismutase and several proteases, suggesting that the cells experience oxidative and proteotoxic stress under inhibitory high CO_2_ levels.

In our study, we used the freshwater cyanobacterium *Synechocystis* sp. PCC 6803 (hereafter referred to as *Synechocystis*), which is a well-characterized model organism (Ikeuchi and Tabata 2001; Zavřel et al. 2017). In order to study which genes are beneficial or detrimental under very high CO_2_ conditions, we have used a clustered regularly interspaced short palindromic repeats (CRISPR) interference (CRISPRi) library of *Synechocystis* constructed as previously described (Yao et al. 2020; Miao et al. 2023). By exposing a culture of this mutant library to either 30% CO_2_ (inhibitory concentration) or 4% CO_2_ (optimal) conditions, we observed which gene knockdowns enhanced or suppressed growth. In all experiments, pH was buffered at around 7.5 using sodium bicarbonate in order to avoid acidification of the medium at high CO_2_ concentrations. Finally, we investigated the growth behavior of wild type (WT) and a mutant strain to confirm predictions made from the CRISPRi experiments.

## Methods

### Strains

We used a glucose-tolerant substrain of *Synechocystis* sp. PCC 6803 as the WT (The Pasteur Culture Collection of Cyanobacteria (PCC), Paris, France). The Δ*cpcG* mutant (Δslr2051::(Km^R^-*codA*)) was also derived from a glucose-tolerant substrain of *Synechocystis* sp. PCC 6803 (CyanoSource, https://cyanosource.ac.uk; Lea-Smith et al. 2016). The small guide RNA (sgRNA) library was constructed as described by Miao et al. (2023). Briefly, the sgRNAs were inserted in the neutral site slr0397 in a glucose-tolerant base strain of *Synechocystis* sp. PCC 6803 with a dCas9 expression cassette (tetR_PL22_dCas9_SpR) integrated at the neutral site *psbA1*/slr1181. The mutant library contained about 21,700 individual mutants with typically five sgRNA per protein-coding and non-coding site.

### Cultivation media and measurements

All *Synechocystis* strains were cultivated in BG-11 media (Stanier et al. 1971) made from a 50 × stock (cat. no. C3061, Sigma, Darmstadt, Germany). For maintenance, liquid cultures were grown at ambient air and temperature (approx. 22 °C) on a rotary shaker (approx. 100 rpm) under a light intensity of about 50 μmol photons m^−2^ s^−1^. As inoculum for experiments with defined CO_2_ exposure, cultures were incubated at 30 °C, 200 µmol photons m^−2^ s^−1^ light intensity, and bubbled with air containing 4% CO_2_. For all experiments with exposure to a gas phase with added CO_2_, the BG-11 media were supplemented with 20 mM HEPES (from a stock of autoclaved 1 M HEPES where pH had been adjusted to 7.5 with NaOH) and with an appropriate amount of NaHCO_3_ (from a stock of filter-sterilized 1 M NaHCO_3_) as described below such that a pH of 7.5 was obtained upon bubbling. Gas mixtures of air and a defined content of CO_2_ were generated by mass flow controller gas mixers (model IGM, Gometrics, Barcelona, Spain; model GMS150, Photon Systems Instruments, Drasov, Czech Republic; model Zephyr, AlyTech, Juvisy sur Orge, France). The gas flow rates were 1 vol gas per 1 vol liquid per minute.

The following approach was used to calculate inorganic carbon concentrations in the cell-free cultivation media. The CO_2_ concentration in the liquid in equilibrium with the gas phase was calculated from the CO_2_ content in the gas phase using Henry’s Law (Weiss 1974), [CO_2_] = *K*_H_ *p*_CO2_, where *K*_H_ = 0.02964 M atm^−1^ and *p*_CO2_ is the CO_2_ partial pressure in the gas phase (e.g., *p*_CO2_ = 0.30 atm at a content of 30% CO_2_). The *K*_H_ value was estimated using the empirical formula by Weiss (1974) and assuming a salinity of 1.5 (based on BG-11 medium with 20 mM HEPES at pH 7.5) and a temperature of 30 °C. The concentrations of HCO_3_^−^ and CO_3_^2−^ in the liquid in equilibrium with the gas phase were calculated using the chemical equilibrium assumptions: *K*_a1_ = [HCO_3_^−^] [H^+^] / [CO_2_] and *K*_a2_ = [CO_3_^2−^] [H^+^] / [HCO_3_^−^]. The equilibrium constants were determined empirically by titration of 20 mM Na_2_CO_3_ with 1 M HCl in an aqueous solution at 30 °C containing the most abundant salts in BG-11 medium (NaNO_3_, 1.5 g L^−1^; MgSO_4_·7H_2_O, 0.075 g L^−1^).

Cell concentrations were determined as the optical density at 730 nm (OD_730_) measured on a UV-1800 spectrophotometer (Shimadzu, Kyoto, Japan). The total inorganic carbon (TIC) concentration in the medium was determined using a TOC-L machine (Shimadzu, Kyoto, Japan) and freshly made NaHCO_3_ solutions as standards. pH was determined using a WTW Sentix 41 electrode (Xylem Analytics GmbH, Weilheim, Germany).

### Growth rate determination of WT and Δ*cpcG* mutant

The growth rates of the WT and Δ*cpcG* mutant were determined at different CO_2_ contents and salinities in order to characterize the response to high CO_2_ and high Na^+^ concentrations. Cultivation was performed in an eight-tube Multi-Cultivator MC-1000-OD photobioreactor (Photon System Instruments, Drasov, Czech Republic) with a culture volume of 50 mL per tube. The temperature was set at 30 °C, and constant light was set at 200 µmol photons m^−2^ s^−1^. The gas bubbling rate was set at 50 mL min^−1^ per tube. The media used was BG-11 with 20 mM HEPES buffer. The pH was adjusted with NaHCO_3_ to be stable at a value of 7.5 when bubbling. During the growth experiments to determine CO_2_ tolerance, cultures were exposed to four different CO_2_ conditions: 0.04% (air, representing carbon limitation), 4% (representing optimal CO_2_ conditions), 15% (representing high CO_2_ conditions), and 30% (representing very high CO_2_ conditions). In the case of the salinity stress experiment, all the cultures were grown at 4% CO_2_ with the addition of various concentrations of NaCl. Cultivation was done in batch mode with an inoculation at OD_730_ of 0.05 from a preculture grown with 4% CO_2_. The growth rate was determined by an exponential fit to the OD_720_ as recorded by the Multi-Cultivator between values of 0.1 and 0.3. The samples were grown in duplicates.

### Turbidostatic cultivation of the CRISPRi mutant library

A preculture of the CRISPRi library (Miao et al. 2023) was revived from a freezer stock and grown in a shaking flask, at 150 rpm, 30 °C, 50–100 µmol m^−2^ s^−1^, and 1% CO_2_. The medium used was BG-11 with 25 mM HEPES, containing 25 µg mL^−1^ of spectinomycin and 25 µg mL^−1^ of kanamycin.

Cultivation of the CRISPRi mutant library under selective conditions was performed in an eight-tube Multi-Cultivator MC-1000-OD photobioreactor (Photon System Instruments, Drasov, Czech Republic) with 65 mL culture volume per tube. All the cultures were inoculated with a starting OD_730_ of 0.1. The temperature was set at 30 °C and the light intensity at 200 µmol photons m^−2^ s^−1^. Temperature, light, and the turbidostat pumping system were controlled by a custom software available at https://gitlab.com/mmp-uva/pycultivator (Du et al. 2016). Cultures were exposed to two different CO_2_ conditions, 4% (representing optimal CO_2_ conditions) and 30% (representing very high CO_2_ conditions). The 4% CO_2_ gas mixture was provided by a GMS150 gas mixing system (Photon System Instruments, Drasov, Czech Republic) and the 30% CO_2_ gas mixture by two Lambda MASSFLOW gas flow controllers (one for air and one for CO_2_; Lambda Instruments, Brno, Czech Republic). The gas bubbling rate was set at 70 mL min^−1^ per tube. Cultivations were performed in quadruplicates, although one 30% CO_2_ culture failed to grow, leaving only triplicates of this condition. The medium used was BG-11 (cat. no. C3061, Sigma, Darmstadt, Germany) with HEPES buffer and the pH was adjusted with NaHCO_3_ to be stable at a value of 7.5 when bubbling as described above.

The turbidity threshold for the turbidostat was set at an OD_720_ of 0.2, being measured every 15 min automatically by the photobioreactor. If this limit was exceeded in three measurements in a row in a tube, the content was diluted with 5 mL of fresh medium. The specific growth rate (*µ*) was calculated continuously by the software from the OD_720_ values recorded by the photobioreactor and the generation time (*T*_gen_) was calculated as *T*_gen_ = ln(2)/*µ*. Cell samples representing generations 0, 4, 8, and 10 were collected for sequencing. Generation zero samples were collected immediately before the cultures were induced with IPTG (1 µg mL^−1^). For the sampling, 12 mL of culture was harvested by centrifugation at 4 °C, 5000 × *g* for 10 min. The supernatant was discarded, and cell pellets were stored at − 20 °C for 1–15 days, until DNA extraction. Cultivations were controlled using the web application ShinyMC (https://github.com/m-jahn/ShinyMC, commit e30d6ac from 5th Sep 2023). ShinyMC was used to calculate growth rates using the setting OD correction = false and otherwise default settings.

### Library preparation, sequencing, and statistical analysis

Genomic DNA extraction and library preparation were performed as described previously (Miao et al. 2023). The multiplexed samples were sequenced on an Illumina NextSeq 2000 system using a NextSeq 2000 P1 kit (Illumina, Inc., San Diego, CA, USA). Sequencing data was processed using the Nextflow pipeline nf-core-crispriscreen (https://github.com/MPUSP/nf-core-crispriscreen, commit e4aad5b from the 11th April 2024) with the following settings deviating from the defaults: As adapter to be trimmed using cutadapt (–three_prime_adapter), the input ^CAGTGATAGAGATACTGGGAGCTA … GTTTTAGAGCTAGAAATAGCAAGTTAAAATAAGGC was given. Reads with a mapping quality below one were discarded (–filter_mapq = 1). DESeq2 (Love et al. 2014) was used for normalization and determination of log_2_ fold changes (log_2_FC) of individual sgRNAs compared to generation 0. Fitness scores of individual sgRNAs were defined as the resulting area under the curve (log_2_FC versus time) and weighted mean fitness scores for individual genes were calculated as the average of the fitness scores of sgRNAs targeting a specific gene, weighted according to correlation among sgRNAs and estimated efficiency (see Miao et al. (2023) for more details). For the calculation of adjusted *p*-values using the Wilcoxon rank-sum test, control sgRNAs included in the CRISPRi library were indicated using –gene_controls “ctrl.” For more details on the pipeline, see Miao et al. (2023) and the pipeline’s documentation. Target-specific adjusted *p*-values for the comparison between the 4% and 30% CO_2_ conditions were calculated based on the fitness scores of the sgRNAs associated with a CRISPRi target using the unpaired Wilcoxon rank-sum test and Benjamini-Hochberg (BH) corrections. The pipeline output was further analyzed using custom R scripts. R scripts and pipeline output are available online (GitHub repositories https://github.com/ute-hoffmann/CO2_cyano_CRISPRi and Zenodo repository doi https://doi.org/10.5281/zenodo.14095552).

The difference in fitness score between 30 and 4% CO_2_ was characterized by an impact score defined as the distance (*d*) between the position of the mutation (*x*_0_, *y*_0_) and the identity line (*y* = *x*) in a plot of the fitness score in 30% CO_2_ versus the fitness score in 4% CO_2_ and where *x*_0_ is the fitness score in 4% CO_2_, and *y*_0_ is the fitness score in 30% CO_2_:

$$d=\frac{{y}_{0}-{x}_{0}}{\sqrt{2}}$$

If *x*_0_ and *y*_0_ are similar, the impact of the gene repression is similar in 4% and 30% CO_2_, and the impact score is close to 0. If *x*_0_ and *y*_0_ are substantially different, the impact of the mutation is substantially different at 30% and 4% CO_2_ and the impact score will be numerically large.

A “combined score” for this comparison was calculated as the product of the impact score and the negative decadic logarithm of the adjusted *p*-value comparing the fitness values of sgRNAs at the different conditions for each targeted gene. A higher combined fitness score indicates a mutation with a significant and substantial impact on growth, as it takes into account both the fitness and the reliability of the data. The combined scores were used to select mutations with reliable and meaningful impact.

### Pathway enrichment analysis and interactive browser

We assessed the impact of CRISPRi knockdown on general physiology with a gene set enrichment analysis (GSEA) of the average weighted fitness in selected pathways (clusterProfiler v4.12.6, BH-adjusted *p*-value < 0.01) (Xu et al. 2024). Databases used were CyanoCyc (https://cyanocyc.org/; provides annotation for 704 genes to 261 pathways), KEGG: Kyoto Encyclopedia of Genes and Genomes (https://www.kegg.jp/), and The Gene Ontology (GO) knowledgebase (https://geneontology.org/).

For visual assessment of the CRISPRi results, we created an interactive genome web browser (https://rth.dk/resources/cyanobacteria/CRISPRi-browser) that shows the position and fitness score of each sgRNA with JBrowse 2 (Diesh et al. 2023). We present the data in the *Synechocystis* sp. PCC 6803 genome annotations corresponding to assembly ASM972v1 relative to the CyanoCyc (downloaded August 1, 2024; Moore et al. 2024), “Kazusa2009 + AG Hess” (a combination of the annotations from Kaneko et al. 1996 and Kopf et al. 2014 supplemented with annotations of small proteins from Mitschke et al. 2011 and Baumgartner et al. 2016), and RefSeq (GCF_000009725.1-RS_2024_07_21). For a concise visual representation, the fitness scores are shown as genomic coverage values, which is the maximum absolute fitness plotted at positions in which multiple guides overlap. The position of all sgRNAs and bigWig files of the fitness scores can be downloaded in the browser.

## Results

### Setup of cultivation conditions with constant pH and different CO_2_ concentrations

Bubbling BG-11 medium with high concentrations of CO_2_ without buffering would cause a dramatic and cell-inhibiting drop in pH. Therefore, prior to inoculation, NaHCO_3_ was added to the BG-11 medium at a concentration calculated based on the CO_2_ content in the gas phase to ensure that the pH was about 7.5 at equilibrium between gas and liquid. To calculate the amount of NaHCO_3_ needed, the CO_2_ concentration in the liquid was calculated from the CO_2_ content in the gas phase using Henry’s Law (see “Methods” for details). Then, the HCO_3_^−^ and CO_3_^2−^ concentrations in the liquid were calculated from the calculated CO_2_ concentration in the liquid and the p*K*_a_ values. The p*K*_a_ values were determined by acid titration of Na_2_CO_3_ in a medium analogous to BG-11 at 30 °C, as p*K*_a1_(CO_2_/HCO_3_^−^) = 6.35 ± 0.01 (*n* = 3) and p*K*_a2_(HCO_3_^−^/CO_3_^2−^) = 10.15 ± 0.02 (*n* = 3) (see “Methods” for details). The carbonate alkalinity (CA = [HCO_3_^−^] + 2[CO_3_^2−^]) was calculated based on the calculated HCO_3_^−^ and CO_3_^2−^ concentrations. Finally, NaHCO_3_ was added in an amount equimolar to the calculated carbonate alkalinity.

To ensure the BG-11 medium supplemented with NaHCO_3_ reached the target pH of approximately 7.5, a BG-11 medium without inorganic carbon was first prepared using BG-11 stock (Sigma C3061) and HEPES (1 M, pH 7.5), and adjusted to pH 7.5 with NaOH (10 M). For use of this medium with a specific gas mixture, a portion of water in this medium was replaced by an equal volume of a sterile NaHCO_3_ stock (1 M), to obtain the carbonate alkalinity calculated as shown in Table 1. The media were then bubbled with the desired gas mixture overnight (0.04%, 4%, 15%, or 30% CO_2_), after which the pH and TIC were measured. The final pH after bubbling was 7.5 ± 0.1 as expected, and the measured TIC concentrations were consistent with the predicted values (Table 1). The results show that this cultivation system allows a setup with predictable pH and concentrations of inorganic carbon. Cell growth caused a minor increase in pH, but at cell concentrations of OD_730_ of less than 0.8, the measured pH increase was less than 0.1 pH units in the cultures with CO_2_ levels of 4% or higher (data not shown).

**Table 1:** Predicted and measured pH and inorganic carbon species in BG-11 medium supplemented with NaHCO_3_ and in equilibrium with gas phases with different CO_2_ contents

CO_2_ content in gas phase	NaHCO_3_ added (mM)	Predicted values					Measured values	
		[CO_2_] (mM)	[HCO_3_^−^] (mM)	[CO_3_^2−^] (mM)	CA (mM)	TIC (mM)	pH	TIC (mM)
0.04% (air)	0.168	0.012	0.167	0.000	0.168	0.180	7.51 ± 0.00	0.3 ± 0.1
4%	16.8	1.186	16.747	0.037	16.822	17.970	7.49 ± 0.00	19.7 ± 0.4
15%	63.1	4.446	62.801	0.141	63.082	67.388	7.45 ± 0.01	79.2 ± 1.9
30%	126.2	8.892	125.602	0.281	126.165	134.775	7.44 ± 0.00	136.3 ± 1.7

Parameters used for calculation of predicted values: pH = 7.5, *K*_H_ = 0.02964 M atm^−1^, p*K*_a1_ = 6.35, p*K*_a2_ = 10.15

### Growth of *Synechocystis* WT with different CO_2_ and NaCl concentrations

**Fig. 1:**
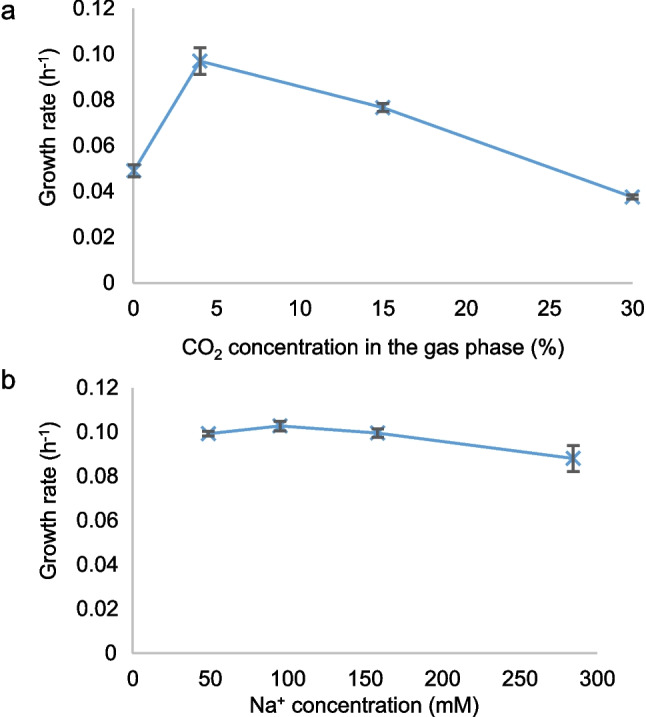
Growth rates of *Synechocystis* WT at a constant pH of about 7.5 and a light intensity of 200 µmol photons m^−2^ s^−1^ (*n* = 2). **a** Growth rates at different CO_2_ contents in the gas phase. The lowest concentration tested was with air (0.04% CO_2_). The pH was maintained by the addition of NaHCO_3_, and hence, the Na^+^ concentration increased with increasing CO_2_ concentration. At 4% and 30% CO_2_, the Na^+^ concentrations were 48.8 and 158.2 mM, respectively. **b** Growth rates at a CO_2_ content of 4% of the gas phase and different Na^+^ concentrations. The Na^+^ concentration was adjusted by the addition of NaCl

Previous experiments have shown that 1–5% CO_2_ is optimal for the growth of *Synechocystis* (Zavřel et al. 2017). We tested the tolerance of *Synechocystis* WT to this and higher levels of CO_2_ by measuring the growth rate under CO_2_ contents of 0.04% (air), 4%, 15%, and 30% of the gas phase while maintaining a pH of approx. 7.5 in the cultivation medium using NaHCO_3_ as described above. The growth rate as a function of the gas phase CO_2_ content is depicted in Fig. 1a. The results show that *Synechocystis* growth was inhibited under CO_2_ contents of 15% or higher. At 30% CO_2_, the growth rate was only 39% of the optimal growth rate at 4% CO_2_. We estimate from the data presented here that the gas phase CO_2_ content at which the growth rate is reduced to half of the growth rate under optimal CO_2_ conditions is approximately 25% CO_2_. In the following experiments, we have used 4% CO_2_ to represent optimal conditions and 30% CO_2_ to represent conditions where the cell growth is significantly inhibited by the high CO_2_ content.

The Na^+^ concentration in the cultivation medium increased with increasing CO_2_ content in the gas phase because NaHCO_3_ was used to maintain pH (see “Results,” first section). In pure BG-11 medium without any additions other than the BG-11 stock solution, the Na^+^ concentration was 18.0 mM. In our BG-11 medium maintained at pH 7.5 using HEPES and NaHCO_3_, the total Na^+^ concentration was 32.2, 48.8, 95.1, and 158.2 mM at 0.04%, 4%, 15%, and 30% CO_2_, respectively (calculated based on the medium composition and the addition of NaHCO_3_). Therefore, the question arises how cells in media with high CO_2_ contents and stable pH are affected by the increased Na^+^ concentration. The growth rate as a function of Na^+^ concentration with a constant gas phase CO_2_ content of 4% is shown in Fig. 1b. We conclude that the growth rate of cells in BG-11 medium supplemented with NaCl to reach the same Na^+^ concentration (158.2 mM) as with 30% CO_2_ was not affected by the added NaCl. Thus, the inhibition of the cells with 30% CO_2_ is likely to be a consequence of elevated concentrations of inorganic carbon in the medium, and not the elevated Na^+^ concentrations due to the addition of NaHCO_3_.

### Phenotype screening under 4% and 30% CO_2_ using a CRISPRi library

#### Assessment of phenotype

**Fig. 2:**
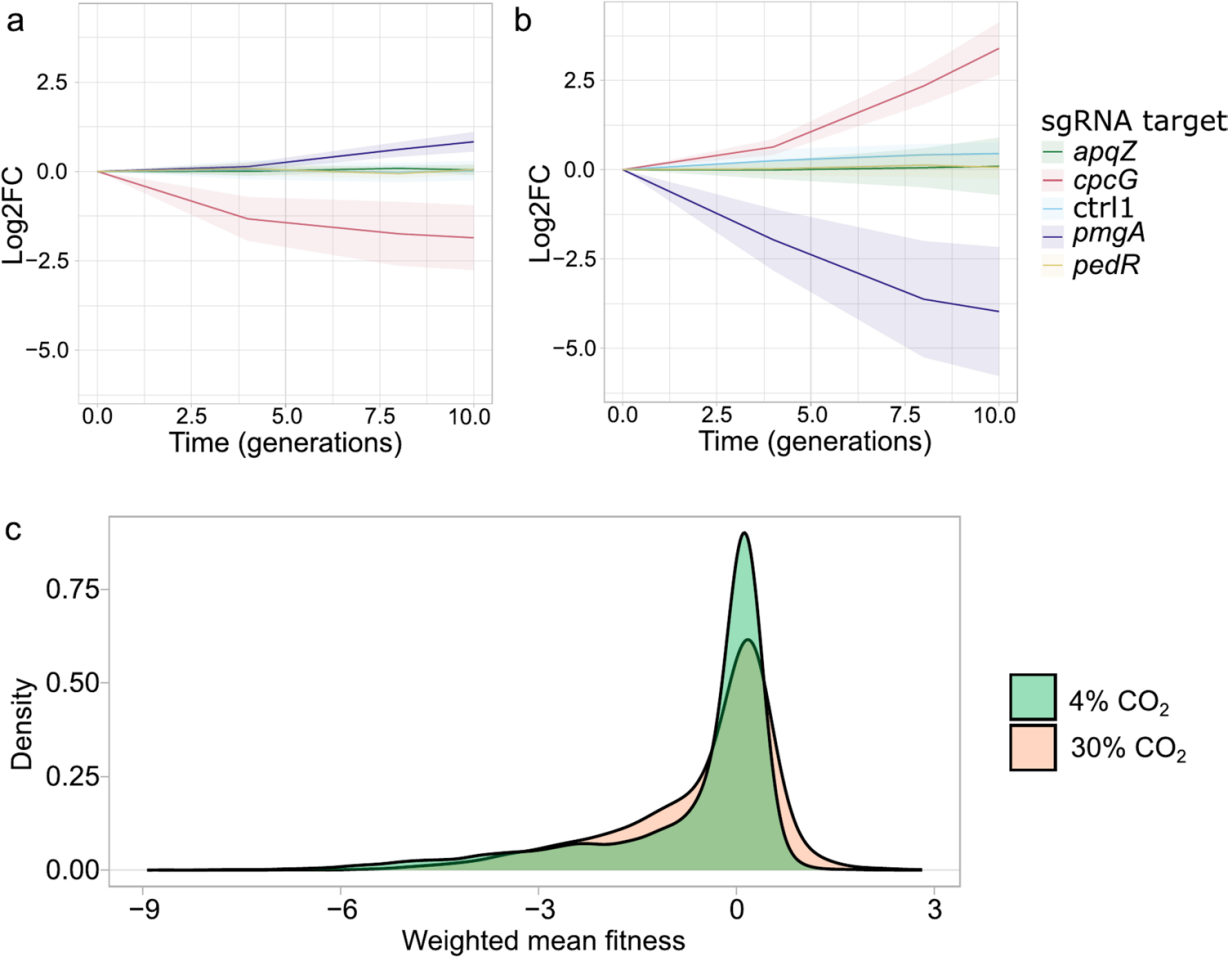
**a** Change in abundance of CRISPRi mutants over ten population generations in 4% CO_2_ of representative genes (*apqZ*/slr2057, aquaporin; *cpcG*/slr2051, phycocyanin linker; ctrl1, control sgRNA 1; *pmgA*/sll1968, photosynthesis regulator; *pedR*/sll0564, PedR transcription factor). Lines represent the weighted mean average of five sgRNAs (sgRNAs that do not correlate well with others targeting the same gene have less weight, see Methods in Miao et al. (2023)); shaded areas give the corresponding 95% confidence interval for this. **b** Change in abundance of the same genes in 30% CO_2_. **c** Distribution of fitness scores for all sgRNA targets represented by the CRISPRi mutant library cultivated in 4% and 30% CO_2_

A CRISPRi repression mutant library of *Synechocystis* was set up in turbidostatic cultivation mode with either 4% or 30% CO_2_ in the gas phase and the cultures were allowed to grow for ten generations. The average growth rate of the cells in 4% CO_2_ over the ten generations was about 0.058 h^−1^, while cells in 30% CO_2_ had a growth rate of about 0.038 h^−1^ (Supplementary Fig. S1). Replicates for the different cultivations correlated well (Supplementary Fig. S2). A fitness score for 3424 putative protein-coding genes and 1686 non-coding loci represented by the CRISPRi library was calculated for the 4% and 30% CO_2_ condition as described previously (Miao et al. 2023; see “Methods” and Supplementary Table S1). Briefly, the fitness scores were derived from the change in relative abundance of each mutant within the population over time (in our case ten generations), thus providing a measure of the relative growth rate of each mutant compared to all other mutants under the same cultivation conditions. Figure 2 shows the abundance of a few selected mutants during the turbidostatic cultivation (Fig. 2a and b) and the distribution of calculated fitness scores of all sgRNA targets tested (Fig. 2c). Examples with other selected relevant genes are shown in Supplementary Fig. S3. The analyzed fitness data for all mutants can be accessed on an interactive web application (https://m-jahn.shinyapps.io/ShinyLib/).

The results showed that CRISPRi repression of about one-third of all protein-coding genes resulted in substantially reduced fitness (here defined as a fitness score of less than − 1) under both conditions tested (4% and 30% CO_2_). This includes genes with essential functions in protein synthesis, replication, and energy metabolism. This observed decrease in fitness is in agreement with repression of essential genes, thus confirming the validity of the CRISPRi experimental model. In stark contrast, much fewer repressions of protein-coding genes exhibited substantially increased fitness (here defined as a fitness score of greater than 1) (Fig. 2c). In 4% CO_2_, only 7 genes (0.2% of all genes) had substantially increased fitness, whereas in 30% CO_2_, it was 86 genes (2.5% of all genes). Table 2 lists the genes that when repressed resulted in the highest fitness under 30% CO_2_. A *p*-value and a combined score were calculated for the fitness and impact scores to assess their statistical significance as described in the “Methods” section (Supplementary Table S2). However, we note that setting low cutoff values for these statistical parameters may not be helpful when evaluating the fitness and impact scores of specific genes because valuable information could be overlooked. Therefore, careful inspection of the data is important. For example, considerations about possible effects of non-coding regions and polycistronic transcripts involving the genes of interest might be relevant.

**Table 2:** CRISPRi repression mutants with the highest fitness scores in 30% CO_2_

Locus	Gene	Gene product	Fitness in 30% CO_2_	Fitness in 4% CO_2_	Impact score
sll8017		Hypothetical protein	2.82	0.02	1.98
slr2051	*cpcG*	PBS rod-core linker polypeptide	2.59	− 2.48	3.59
sll1051	*cpcF*	Phycocyanin alpha-subunit phycocyanobilin lyase	2.52	− 1.00	2.49
sll0269		Hypothetical protein	2.40	− 0.07	1.74
sll1579	*cpcC2*	PBS rod linker polypeptide	2.28	− 1.71	2.82
sll1580	*cpcC1*	PBS rod linker polypeptide	2.27	− 2.04	3.05
slr1125		Probable glucosyl transferase	2.25	− 0.32	1.82
slr8021		Hypothetical protein	2.21	− 0.37	1.83
sll0268		Hypothetical protein	2.15	0.04	1.49
slr1042	*pilH*	Two-component response regulator CheY subfamily	2.14	1.74	0.29

**Fig. 3:**
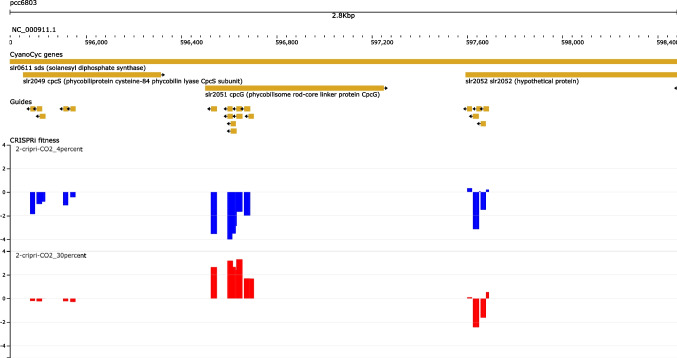
An example of the interactive browser (https://rth.dk/resources/cyanobacteria/CRISPRi-browser) visualizing a section of the *Synechocystis* genome encoding the PBS rod-core linker *cpcG*/slr2051 and the genomic neighboring genes according to their CyanoCyc annotated positions (first track). The genomic positions of the sgRNA sequences are shown below the genes (second track). The track below indicates the weighted mean fitness of each guide in the 4% CO_2_ (blue) and 30% CO_2_ (red) condition as a coverage track. For positions in which two or more guides overlap, the coverage track shows the fitness score for the guide with the maximal absolute value

An interactive browser that maps all the individual sgRNA and their experimentally determined fitness scores onto the *Synechocystis* genome was constructed (Fig. 3; available at https://rth.dk/resources/cyanobacteria/CRISPRi-browser). This browser incorporates the annotation of protein-coding genes, non-coding RNAs, and transcriptional units and helps to visualize where the sgRNAs are located with respect to the genes and their transcriptional environment. An example is shown in Fig. 3, which clearly shows that all the sgRNA targeting *cpcG*/slr2051 caused negative fitness in 4% CO_2_ and positive fitness in 30% CO_2_.

**Fig. 4:**
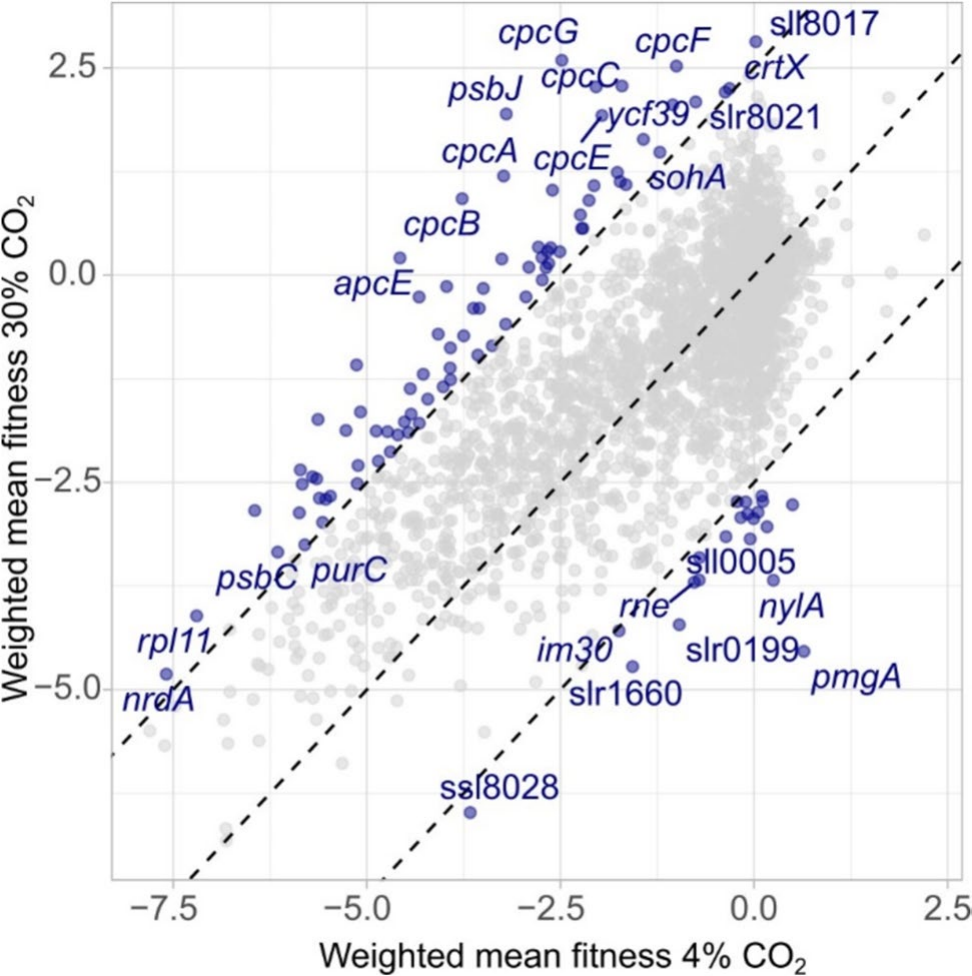
Dot plot of fitness under 30% CO_2_ versus fitness under 4% CO_2_ of CRISPRi knockdown mutations of all protein-coding genes. Dashed lines correspond to the identity line and parallel lines with an intercept of 2.5 and − 2.5

In order to visualize the fitness distribution of mutants cultivated in 4% and 30% CO_2_, the fitness score of all mutants under 30% CO_2_ was plotted against the fitness score under 4% CO_2_ (Fig. 4 and Supplementary Fig. S4). In Fig. 4, the impact score of a gene equals the distance from the gene to the identity line and is an expression of the difference in fitness between the two conditions. The gene with the highest impact score is *cpcG*/slr2051 (3.6), while *pmgA*/sll1968 has the lowest impact score (− 3.7). Table 3 lists the genes that when repressed resulted in the highest impact score. The interpretation is that a numerically large positive impact score indicates that the mutation exhibited better growth under 30% than under 4% CO_2_ and a numerically large negative impact score indicates that the mutation exhibited worse growth under 30% than under 4% CO_2_. In order to investigate which gene repressions caused increased cellular tolerance to very high CO_2_ levels, we need to examine genes that either had a high fitness score in 30% CO_2_ or had a large positive impact score.

**Table 3:** CRISPRi repression mutants with the highest and lowest impact scores

Locus	Gene	Gene product	Fitness in 30% CO_2_	Fitness in 4% CO_2_	Impact score
smr0008	*psbJ*	Photosystem II PsbJ protein	1.95	− 3.20	3.64
slr2051	*cpcG*	PBS rod-core linker polypeptide	2.59	− 2.48	3.59
slr0335	*apcE*	PBS core-membrane linker polypeptide	0.21	− 4.57	3.38
sll1577	*cpcB*	Phycocyanin beta subunit	0.92	− 3.77	3.32
sll1578	*cpcA*	Phycocyanin alpha subunit	1.20	− 3.23	3.13
sll1580	*cpcC1*	PBS rod linker polypeptide	2.27	− 2.04	3.05
sll1816	*rps13*	30S ribosomal protein S13	− 0.26	− 4.33	2.87
sll0711	*ispE*	Isopentenyl monophosphate kinase	− 1.08	− 5.13	2.86
sll1579	*cpcC2*	PBS rod linker polypeptide	2.28	− 1.71	2.82
sll0849	*psbD1*	Photosystem II reaction center D2 protein	− 1.74	− 5.63	2.75
sll0306	*sigB*	RNA polymerase group 2 sigma factor B	− 2.86	0.05	− 2.06
sll1504		Hypothetical protein	− 2.94	− 0.01	− 2.07
slr1129	*rne*	Ribonuclease E	− 3.70	− 0.77	− 2.07
sll0005		Hypothetical protein	− 3.68	− 0.71	− 2.10
ssl3364	*cp12*	CP12 polypeptide	− 3.18	− 0.05	− 2.21
slr1660		Hypothetical protein	− 4.72	− 1.57	− 2.23
slr0195		Hypothetical protein	− 3.03	0.17	− 2.26
slr1916		Probable esterase	− 2.77	0.50	− 2.31
sll0828	*nylA*	Putative amidase	− 3.68	0.25	− 2.78
sll1968	*pmgA*	Photomixotrophic growth-related protein	− 4.54	0.64	− 3.66

**Fig. 5:**
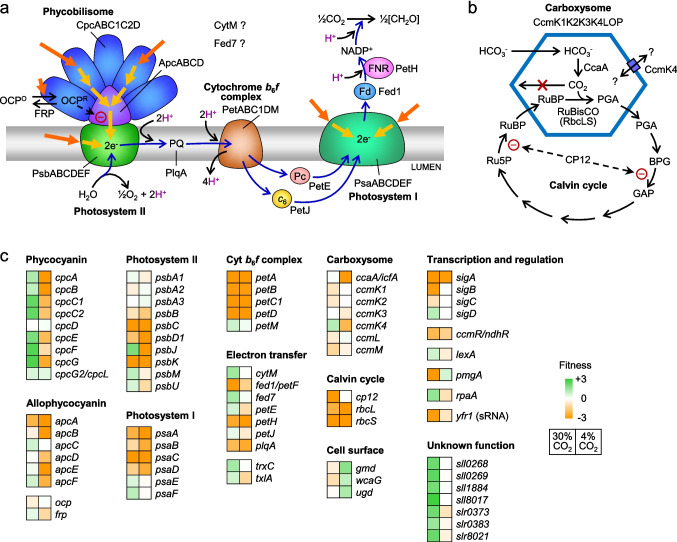
Metabolic pathways in *Synechocystis* of **a** light harvesting and photochemical electron transfer and **b** CO_2_ fixation. **c** Heatmap-visualized fitness scores of the CRISPRi screen of genes encoding these pathways and other genes discussed in the text. Abbreviations: *c*_6_, cytochrome *c*_6_; Fd, ferredoxin; FNR, ferredoxin-NADP.^+^ reductase; FRP, fluorescence recovery protein; OCP, orange carotenoid protein; Pc, plastocyanin; PQ, plastoquinone-9

Gene set enrichment analyses (GSEA) were performed using pathways from different databases (Supplementary Fig. S5; see “Methods” for details). These analyses especially pointed at the repression of PBS as highly significant for growth enhancement in 30% CO_2_. A graphical representation of metabolic pathways and genes where CRISPRi repression resulted in either numerical high fitness scores in 30% CO_2_ or very different fitness scores in 4% and 30% CO_2_ are summarized in Fig. 5. Results with specific genes and physiological activities are presented in the following.

#### Light energy harvest and transfer

The strongest growth-improving phenotype in 30% CO_2_ among all CRISPRi repression mutants was observed among genes encoding structural components of phycocyanin (PC), specifically *cpcG*/slr2051 (also known as *cpcG1*) and the polycistronic *cpcBAC2C1*/sll1577–sll1580, as well as genes involved in the biogenesis of PC, specifically the *cpcE*/slr1878 and *cpcF*/sll1051 genes (Zhang et al. 2014). Deletion mutants lacking these genes have previously been shown to either lack or have functionally reduced PC and PBS (Zhao et al. 2001; Ughy and Ajlani 2004; Kondo et al. 2005; Lea-Smith et al. 2014; Zhang et al. 2014). These studies also showed that cells with a diminished PC antenna are less prone to photoinhibition. CRISPRi repression of *cpcD*/ssl3093 had no effect on fitness, in agreement with previous observations that a *cpcD* deletion mutant was indistinguishable from WT (Ughy and Ajlani 2004). Growth improvement was also observed with CRISPRi repression of genes encoding allophycocyanin (APC) subunits (*apcE*/slr0335, *apcF*/slr1459, *apcC*/ssr3383). This suggests that a smaller PBS antenna was advantageous under the very high CO_2_ in our growth conditions and that the WT cannot downregulate the transcription of *cpc* and *apc* genes sufficiently to avoid photoinhibition.

The CRISPRi *cpcL*/sll1471 (also known as *cpcG2*) repression mutants did not have a strongly affected fitness in neither 4% nor 30% CO_2_. CpcG and CpcL are homologous, but CpcG is a component of the conventional PBS supercomplex that normally is associated with PSII and is required for state transitions, whereas CpcL-containing PBS are not involved in state transition (Kondo et al. 2005, 2009) but is part of a core-free PBS that is specific for PSI (Watanabe et al. 2023).

The orange carotenoid protein (*ocp*/slr1963) functions in protection from photoinhibition by being activated from an inactive state, OCP^O^, to an active state, OCP^R^, under high light (Kirilovsky and Kerfeld 2016). The active state binds to the PBS, inducing thermal energy dissipation, and thereby reduces PBS fluorescence and energy transfer. The fluorescence recovery protein (*frp*/slr1964) reverts this quenching by accelerating the conversion of OCP^R^ back to the inactive OCP^O^ state (Fig. 5a). CRISPRi repression of *ocp* did not affect growth under 4% CO_2_ but resulted in a minor growth suppression under 30% CO_2_, indicating that non-photochemical quenching by OCP is advantageous under very high CO_2_ conditions. In contrast, repression of *frp* was detrimental to growth under 4% CO_2_ but beneficial under 30% CO_2_. This suggests that the absence of FRP results in prolonged activity of OCP^R^, thus promoting non-photochemical quenching of the PBS. Such sustained quenching could be disadvantageous under optimal growth conditions (4% CO_2_) but appeared to be advantageous under 30% CO_2_, where the cells appear to experience photoinhibition. This positive effect on growth by reduced light harvesting under very high CO_2_ is similar to the effect observed with the CRISPRi repression of *cpc* genes presented above.

Even though reduction of the PBS antenna appeared to confer a positive fitness (see above), CRISPRi repression of the genes *nblA1*/ssl0452 and *nblA2*/ssl0453 encoding PBS degradation proteins had no significant effect on fitness, presumably because these genes are not transcribed under the nitrate-replete conditions used in our experiments (Baier et al. 2001).

Consistent with our findings, Miao et al. (2023) reported that CRISPRi repression of *cpc*, *apc*, and *frp* genes under inhibitory high light conditions (1000 μmol photons m^−2^ s^−1^) enhances growth compared to intermediate and low light conditions (150 and 60 μmol photons m^−2^ s^−1^, respectively). These, and our results, suggest that cells with a functionally reduced PBS antenna gain a growth advantage in 30% CO_2_ by alleviating photoinhibition. This advantage may be particularly pronounced under our cultivation conditions (and those of Miao et al. 2023) due to the relatively low culture densities used (OD_730_ ≈ 0.2).

#### Photochemical electron transfer

CRISPRi repression of a few genes encoding subunits of PSII (notably *psbJ*/smr0008) and PSI (notably *psaF*/sll0819) improved growth under 30% CO_2_. A *psbJ* deletion mutant of *Synechocystis* has functional PSII, but the PSII complexes have decreased photochemical electron transfer efficiency (Regel et al. 2001). PsaF is likewise a dispensable subunit for PSI (Chitnis et al. 1991). PsaF is involved in the docking of plastocyanin, and hence, its absence may slow electron transfer from plastocyanin to PSI. CRISPRi repression of either plastocyanin (*petE*/sll0199) or cytochrome *c*_6_ (*petJ*/sll1796, previously known as cytochrome *c*_553_) had only little inhibitory effect on growth under 4% CO_2_, probably because these electron carriers can substitute for each other in electron transfer from cytochrome *b*_6_*f* to PSI (Fig. 5a), but under 30% CO_2_, repression of *petE* and *petJ* resulted in a minor growth enhancement. Overall, these observations indicate that a minor suppression of light-driven electron transfer reactions conferred a growth-enhancing phenotype under 30% CO_2_.

In contrast, CRISPRi repression of *plqA*/slr0926, which is essential for plastoquinol-9 biosynthesis, was detrimental for growth under both 4% and 30% CO_2_, in agreement with that there is no known alternative for the electron-transferring function of plastoquinone. Likewise, repression of *petF*/ssl0020 (also known as *fed1*), which encodes the most abundant ferredoxin in *Synechocystis*, also strongly suppressed growth in both conditions, consistent with PetF being essential for growth.

Among the genes with less well-known functions that appear to encode redox-active proteins and potentially regulate the photosynthetic machinery and CO_2_ concentrating mechanisms are *petF7*/sll0662 (also known as *fed7*; Mustila et al. 2014), *cytM*/sll1245 (cytochrome *c*_M_; Solymosi et al. 2020), *petM*/smr0003 (cytochrome *b*_6_*f* complex subunit PetM; Schneider et al. 2001), *trxC*/sll1057 (thioredoxin C; Pérez-Pérez et al. 2009), and *txlA*/sll1980 (thiol:disulfide interchange protein). The observation that CRISPRi repression of these genes enhanced growth under 30% CO_2_ suggests that, if these genes indeed participate in regulatory pathways, these pathways are not optimized for growth under very high CO_2_ conditions and may, in fact, be counterproductive.

#### CO_2_ uptake and fixation

Many genes in *Synechocystis* are involved in CO_2_ concentrating mechanisms (CCM) that actively capture CO_2_ and HCO_3_^−^ when the environmental levels of inorganic carbon are low (Hagemann et al. 2021). Most of these genes are downregulated under high CO_2_ conditions (Hagemann et al. 2021; Kurkela and Tyystjärvi 2024). The statistical significance of fitness scores calculated for most of these genes in our experiments (genes encoding BCT1, BicA, SbtA, NDH-I_3_, and NDH-I_4_) was low, probably in part because the expression levels of these genes were already low under the high CO_2_ conditions in our experiments, as they are repressed by cellular regulatory mechanisms. Therefore, CRISPRi repression of these genes likely had minimal impact on growth rate.

This native repression of CCM genes under high CO_2_ is controlled in part by the transcription factors CcmR(NdhR)/sll1594 and CyAbrB2/sll0822 (Hagemann et al. 2021; Kurkela and Tyystjärvi 2024). Deletion of *ccmR* or *cyabrB2* in *Synechocystis* has been shown to increase CCM gene expression levels even under 3–5% CO_2_ (Wang et al. 2004; Lieman-Hurwitz et al. 2009). This appears to be disadvantageous for growth in high CO_2_ because, in our experiments, CRISPRi repression of *ccmR* or *cyabrB2* caused growth suppression under both 4% and 30% CO_2_. This is probably because the cells are saturated with inorganic carbon and therefore may suffer from activation of CCM. A similar explanation may apply to the CRISPRi repression of the non-coding, small RNA Yfr1/ncr1525 (66 nucleotides), which also caused reduced fitness under 30% CO_2_. Yfr1 has been reported to target *sbtA* mRNA, and a *yfr1* deletion mutant of *Synechocystis* has been shown to have high levels of *sbtA* mRNA and reduced growth under high CO_2_ conditions (Nakamura et al. 2007). Together, these findings support the idea that de-repression of the active CO_2_ and HCO_3_^−^ uptake mechanisms is unfavorable for growth under very high CO_2_ conditions, presumably because the intracellular concentration of inorganic carbon is already very high.

CRISPRi repression of a few components of the CO_2_ fixation machinery improved growth under 30% CO_2_ when compared to 4% CO_2_, notably repression of *ccmK4*/slr1839 and *ccaA*/slr1347 (also known as *icfA*). CcmK4 is a component of the carboxysome shell and may regulate the exchange of metabolites between the cytosol and carboxysome interior (Sommer et al. 2019). CcaA is a carboxysomal carbonic anhydrase that enhances the rate of HCO_3_^−^ conversion to CO_2_ inside the carboxysome and thus enhances the rate of CO_2_ fixation (Hagemann et al. 2021). CRISPRi repression of genes encoding other key carboxysome components had minimal effect on growth under both 30% and 4% CO_2_. This suggests that the knockdown of carboxysome components that enhance substrate availability for CO_2_ fixation may be advantageous under 30% CO_2_. A possible explanation for this is that excessive CO_2_ fixation activity contributes to growth inhibition under these conditions. In agreement with this idea, CRISPRi repression of *cp12*/ssl3364 greatly decreased fitness under 30% CO_2_. CP12 is a repressor of the Calvin cycle that is activated in dark and oxidizing conditions and inactivated in light and reducing conditions (Lucius and Hagemann 2024). Therefore, it is expected that CP12 should be inactive under optimal photoautotrophic conditions, and apparently, it was, since CRISPRi repression had no effect under 4% CO_2_ (Fig. 5c). However, the growth inhibition observed with CRISPRi repression of CP12 under 30% CO_2_ supports the idea that CP12 was activated under these conditions and that CP12-mediated reduction of the rate of CO_2_ fixation was beneficial for growth under 30% CO_2_. This unexpected activation of CP12 under photoautotrophic conditions could be mediated by high concentrations of metabolites that may accumulate as a result of excess CO_2_ fixation in 30% CO_2_ because it appears that certain catabolic products of glucose in cyanobacteria also activate CP12 (Lucius and Hagemann 2024).

With respect to carbon storage compounds, there was no statistically significant difference between 30 and 4% CO_2_ when genes involved in glycogen and polyhydroxyalkanoate metabolism were repressed. This suggests that any differences in balancing of the carbon metabolism at 30% and 4% CO_2_ did not involve these important intracellular carbon pools.

#### Nitrogen metabolism

Cells were cultivated with nitrate as the sole source of nitrogen. It has been established that *Synechocystis* under these conditions expresses the high-affinity Amt1 NH_4_^+^ uptake transporter (*amt1*/sll0180), probably to recapture ammonium lost by passive diffusion of NH_3_ out of the cell (Montesinos et al. 1998; Ohashi et al. 2011). Surprisingly, CRISPRi repression of *amt1* strongly promoted growth under 30% CO_2_ but not under 4% CO_2_ (Supplementary Fig. S6), suggesting that NH_4_^+^ recapture was not beneficial under 30% CO_2_. CRISPRi repression of a few other non-essential genes involved in NH_4_^+^ assimilation (notably *glsF*/sll1499 encoding ferredoxin-dependent glutamate synthase) also affected growth differently under 4% and 30% CO_2_ (Supplementary Fig. S6). This suggests that NH_4_^+^ metabolism was suboptimally regulated under 30% CO_2_, which may be linked to the possible imbalance in carbon metabolism downstream of the Calvin cycle as described above.

#### Poorly known regulatory systems

The group 2 sigma factors SigB and SigD are involved in stress responses and in the transcription of a subset of genes involved in photosynthesis (Montgomery 2014). CRISPRi repression of *sigB*/sll0306 and *sigD*/sll2012 did not affect fitness significantly under 4% CO_2_ but had opposing effects under 30% CO_2_. This aligns with that SigB contributes to a beneficial downregulation of photosynthetic capacity under stress conditions and that SigB and SigD act antagonistically (Imamura et al. 2003).

The strongest growth-suppressing phenotype among all CRISPRi repression mutants in 30% CO_2_ compared to 4% CO_2_ was that of *pmgA*/sll1968. PmgA is important for downregulation of the photochemical systems in *Synechocystis* under high light (Hihara et al. 1998; Muramatsu et al. 2009). We also note that the *pmgA* and slr1916 mutants behaved very similarly in our system (Table 3) in agreement with previous findings that deletion mutants lacking these genes had both an increased PSI/PSII ratio and an increased glucose sensitivity (Ozaki et al. 2007) and that CRISPRi repression mutants targeting these genes had a similar growth-enhanced phenotype under photoautotrophic conditions (Yao et al. 2020). Thus, our results show that *pmgA* and slr1916 were important for adaptation to very high CO_2_ conditions, probably by adjusting the photosynthetic machinery.

In contrast, CRISPRi repression of other regulators resulted in a growth-stimulating phenotype under 30% CO_2_. This includes repression of *rpaA*/slr0115, a response regulator of genes involved in photosynthesis and carbon metabolism (Köbler et al. 2018), and *lexA*/sll1626, a regulator involved in inorganic carbon assimilation (Domain et al. 2004). These findings suggest that certain cellular regulatory systems do not function optimally for growth under very high CO_2_ conditions and may, in fact, be counterproductive.

#### Cell surface structures

Extracellular appendages called pili are found in most cyanobacteria, including *Synechocystis*, and are involved in many different functions, including motility, adhesion, biofilm formation, metal ion acquisition, and DNA uptake (Schuergers and Wilde 2015). The *pilH*/slr1042 gene encodes a response regulator, and previous work has shown that a *pilH* deletion mutant of *Synechocystis* exhibits hyperpiliation (Yoshihara et al. 2002). CRISPRi repression of *pilH* resulted in a substantial growth enhancement under both 30% and 4% CO_2_ (Table 2). The major pilin subunit in *Synechocystis* is encoded by *pilA1*/sll1694 and a *pilA1* deletion mutant is devoid of all thick pili (Bhaya et al. 2000). CRISPRi repression of *pilA1* also resulted in growth enhancement under 30% CO_2_. It is not easy to explain how both a supposed increase (in the case of *pilH*) and a supposed reduction (in the case of *pilA1*) of piliation in *Synechocystis* may cause enhanced growth in our CRISPRi experiments under 30% CO_2_, but the results consistently suggest that modification of cell surface structures may be beneficial for growth.

Fucose constitutes about 10% of the sugars in the exopolysaccharides (EPS) that are located on the outside of the *Synechocystis* cells and are important for adaptation to environmental stress (Fisher et al. 2013). Fucose is also part of myxoxanthophyll in *Synechocystis* (Mohamed et al. 2005). This fucose is synthesized by enzymes encoded by *gmd*/sll1212 (GDP-mannose 4,6-dehydratase) and *wcaG*/sll1213 (GDP-l-fucose synthase). Compared to the WT, a *wcaG* deletion mutant has reduced growth in air and the cell wall and surface do not develop normally (Mohamed et al. 2005). In our experiments, CRISPRi repression of *gmd* and *wcaG* was beneficial for growth under 4% CO_2_, maybe because EPS synthesis is metabolically expensive, yet not promoting growth under this condition, and therefore, less EPS synthesis could result in increased growth by freeing up metabolic resources for other cellular functions. In contrast, CRISPRi repression of *gmd* and *wcaG* was detrimental for growth under 30% CO_2_. The same growth pattern under 4% and 30% CO_2_ was also observed for another gene putatively associated with EPS synthesis, *ugd*/slr1299 (UDP-glucose dehydrogenase) (Xu et al. 2018). This suggests that EPS contributes to optimal growth under 30% CO_2_, consistent with previous experiments that suggest EPS serves as an important carbon sink under high CO_2_ conditions (Kamennaya et al. 2015).

#### Genes with unknown functions and high impact scores

CRISPRi repression of several genes with unknown functions resulted in high fitness under 30% CO_2_ but minimal or no impact on fitness under 4% CO_2_. Prominent examples were sll0268, sll0269, sll1884, sll8017, slr0373, slr0383, and slr8021 (Fig. 5c), all of which have homologs in other cyanobacteria. These genes are promising targets for understanding the physiology of *Synechocystis* under very high CO_2_ conditions because their inactivation could potentially promote growth under such conditions without affecting growth under CO_2_ levels that are optimal for the WT (1–5% CO_2_).

### Testing the prediction that growth inhibition under 30% CO_2_ is light dependent

**Fig. 6:**
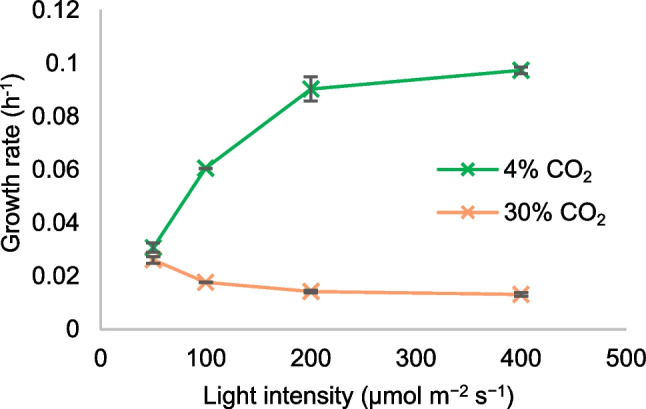
Growth rates of *Synechocystis* WT at different light intensities under optimal CO_2_ (4%, green) and very high CO_2_ (30%, orange) (*n* = 2)

The results described above suggest that the growth of *Synechocystis* with 30% CO_2_ is inhibited by high light. To test this hypothesis, the growth rate of the WT was tested with different light intensities and with either 4% or 30% CO_2_. The results showed that with 4% CO_2_, the cells were not significantly light-stressed within the range tested (up to 400 µmol m^−2^ s^−1^), but that with 30% CO_2_, cell growth was inhibited at light intensities higher than 50 µmol m^−2^ s^−1^ (Fig. 6).

Growth inhibition at high light intensities is typically due to the inability of the cells to dissipate excess absorbed light energy in a harmless manner (Bailey and Grossman 2008; Giacometti and Morosinotto 2013), and therefore, it is expected that a smaller light-harvesting antenna is beneficial under high light stress. This is consistent with the observation that CRISPRi knockdown mutants with a diminished PBS antenna (*cpc* and *apc* genes) was beneficial under 30% CO_2_ (Table 3 and Fig. 5).

### Testing the prediction that knockout of *cpcG* is beneficial under 30% CO_2_

The CRISPRi screening showed that knockdown of *cpc* genes resulted in some of the strongest growth-enhancing phenotypes in 30% CO_2_ (Fig. 5a, Table 3). The *cpc* genes encode PC, which is part of the PBS and one of the main light-harvesting antenna complexes in *Synechocystis*. This suggests that a diminished PC antenna is beneficial for growth under very high CO_2_ conditions. In order to test this hypothesis, we investigated a single-gene mutant lacking *cpcG*/slr2051, corresponding to the CRISPRi *cpc* mutant that showed the highest fitness under 30% CO_2_. CpcG is a non-pigmented polypeptide that links PC and APC in the PBS and is essential for optimal light harvesting (Bryant 1991).

**Fig. 7:**
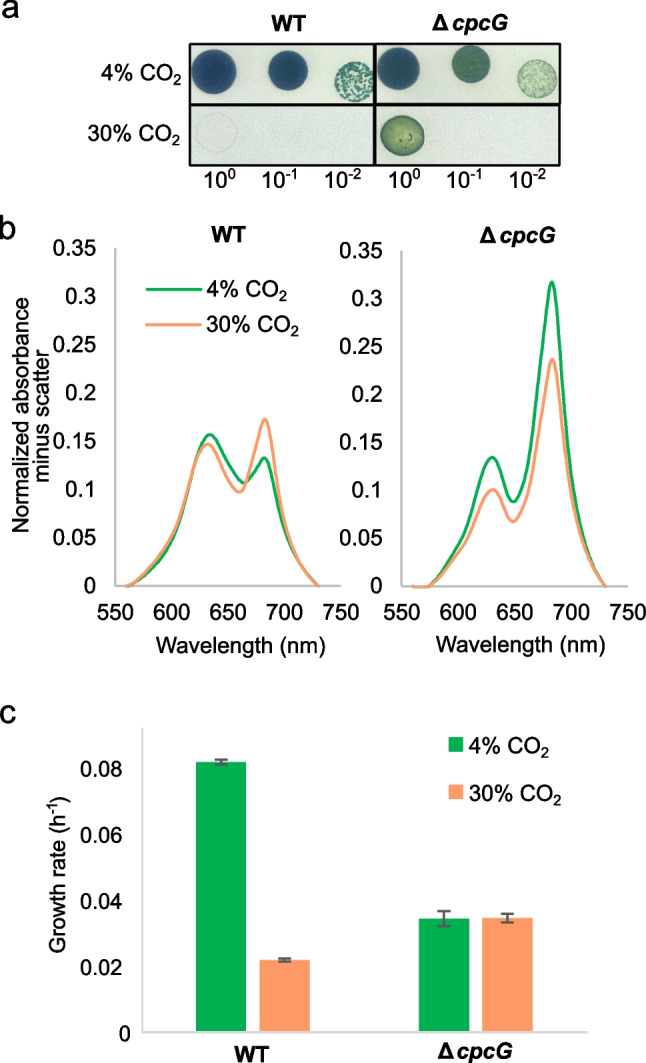
**a** Spot tests of *Synechocystis* WT and Δ*cpcG* mutant under optimal (4%) and very high (30%) CO_2_ conditions. Cultures were grown under 4% CO_2_ conditions until exponential phase, harvested, and diluted with fresh medium to an OD_730_ of 0.3, 0.03, and 0.003 (relative concentrations are shown in the figure, where 10^0^ corresponds to an OD_730_ of 0.3). Then, 6 μL of cell suspension was spotted on BG-11 plates buffered with NaHCO_3_ at pH 7.5 and incubated under either 4 and 30% CO_2_ at 30 °C and 100 μmol photons m^−2^ s^−1^. **b** Normalized absorption spectra of WT and Δ*cpcG* mutant from liquid cultures with either 4% or 30% CO_2_. The absorption spectra were normalized at 730 nm (OD_730_ = 1) and the estimated light scatter (a straight line between OD_560_ and OD_730_ in the spectra) due to the cells was subtracted (Möllers et al. 2014). **c** Growth rates of WT and Δ*cpcG* mutant in liquid culture with 4% (green) or 30% CO_2_ (orange) and a light intensity of 200 µmol m^−2^ s.^−1^ (*n* = 2). For growth curves and growth rate determination, see Supplementary Fig. S7

Figure 7a shows a spot test on solid medium comparing WT and the Δ*cpcG* mutant under 4% and 30% CO_2_ after 6 days of growth. The density of growth in the spot tests suggested that indeed inactivation of *cpcG* resulted in faster growth than the WT under 30% CO_2_. The spot test also showed that the color of the Δ*cpcG* mutant in 4% CO_2_ was slightly different from that of the WT, suggesting that the content of chlorophyll or PC, or both, was different.

Absorption spectroscopy of cells grown in liquid culture showed that the contents of PC and chlorophyll per cell in the WT were similar under 4% and 30% CO_2_ (Fig. 7b), suggesting that the cells cannot substantially alter the levels of these pigments under very high CO_2_. The content of PC per cell appeared to be similar in the WT and the Δ*cpcG* mutant (Fig. 7b), in agreement with previous results conducted using WT and a *cpcG*/SYNPCC7002_A0811 mutant of *Synechococcus* sp. PCC 7002 (Bryant 1991). However, we noticed that the Chl *a* content per cell was higher in the mutant, resulting in a lower PC-to-Chl *a* ratio in the mutant compared to the WT as previously observed (Kondo et al. 2005; Oh and Montgomery 2019). Even though the content of PC per cell appeared to be roughly similar in the WT and the Δ*cpcG* mutant (Fig. 7b), it has previously been shown that PC in a *cpcG* mutant is not effectively assembled onto PBS cores and is not effectively coupled to the Chl antenna of PSII (Bryant 1991).

Cultivation in liquid medium of the WT and Δ*cpcG* mutant showed that the mutant grew slower (*µ* = 0.034 ± 0.002 h^−1^) than the WT (*µ* = 0.081 ± 0.001 h^−1^) with 4% CO_2_ (Fig. 7c), in agreement with the lower fitness score obtained from the CRISPRi screening with 4% CO_2_ (Fig. 3) and in agreement with previous studies (Kondo et al. 2005). However, the Δ*cpcG* mutant grew faster (*µ* = 0.034 ± 0.001 h^−1^) than the WT (*µ* = 0.022 ± 0.001 h^−1^) under 30% CO_2_, again in agreement with the higher fitness score obtained from the CRISPRi screening. Interestingly, the Δ*cpcG* mutant had very similar growth rates with 4% and 30% CO_2_ (Fig. 7c), implying that the growth of the mutant was not inhibited by 30% CO_2_. This supports our hypothesis that a disrupted light-harvesting antenna is beneficial under 30% CO_2_.

## Discussion

This study aimed to identify genes and metabolic pathways in *Synechocystis* that are important for adaptation to inhibitory high CO_2_ concentrations. The CRISPRi-based genome-wide screening employed here provided valuable insights, even with its limitations. While CRISPRi offers high-throughput capability, the nature of variable gene repression, potential off-target interactions, and sometimes contradictory behavior of sgRNA with the same target limit definitive conclusions. In order to provide more statistically robust interpretations, the current CRISPRi library employed five sgRNAs per target. Overall, the CRISPRi methodology allowed us to point out specific genes that influenced growth under high CO_2_, propose hypotheses, and design experiments to test these hypotheses. Our results with CRISPRi and follow-up experiments with WT and mutant cells indicate that modifications in light harvesting, photochemical electron transfer, inorganic carbon management, and several poorly known and unknown systems are essential. Although the cellular regulatory machinery provides some adaptation to inhibitory high CO_2_ (as evidenced by growth suppression under 30% CO_2_ but not under 4% CO_2_ when certain genes, such as *pmgA*, *cp12*, and *sigB*, were repressed by CRISPRi), this does not seem to allow full adaptation to inhibitory high CO_2_ concentrations because in some cases, CRISPRi repression enhanced growth under 30% CO_2_ (e.g., repression of genes involved in light harvesting, photochemical electron transfer, carboxysome structure, and several genes with unknown functions), suggesting that these genes counteract optimal adaptation under inhibitory high CO_2_ conditions.

Our experimental setups used relatively low cell densities (OD_730_ ≈ 0.1–0.3) for growth rate measurements and selection of the CRISPRi library. This approach was primarily chosen to minimize the effects of light limitation and self-shading and to allow comparison with previous studies (Yao et al. 2020; Miao et al. 2023). Photoinhibition is more likely to occur at these low OD conditions than at higher OD conditions. Therefore, our conditions, which were not limiting for light or inorganic carbon, provided an optimal setup for observing the effects of photoinhibition and inorganic carbon on cellular physiology under very high CO_2_ conditions.

Indeed, light harvesting seemed to have a central role in inhibiting cell growth under very high CO_2_. One of the strongest findings was that repression of the PBS antenna under intermediate light conditions was beneficial under 30% CO_2_ while it was not under 4% CO_2_. This relates especially to the main components of the PBS antenna, the PC and APC, and the anchoring protein CpcG. Sauer et al. (2001) showed that cells with a reduced PC and APC antennae were less affected by photoinhibition since PSII activity saturates at significantly higher light intensities. Miao et al. (2023) also concluded that repression of PC and APC in high light was beneficial for the growth of *Synechocystis*. This aligns with the idea that cells growing in low-density cultures and 30% CO_2_ are photoinhibited, since in our study CRISPRi mutants with repression of genes encoding PC (*cpcA*, *cpcB*, *cpcC1*, *cpcC2*) and APC (*apcE*, *apcF*, *apcC*) had improved growth under 30% CO_2_ (Fig. 5). With respect to the CpcG protein, a Δ*cpcG* mutant exhibits a higher content of PSII compared to the WT (Kondo et al. 2005), but displays a defect in energy transfer between the PBS and PSII (Bryant 1991; Kondo et al. 2005; Oh and Montgomery 2019). Furthermore, the disruption of *cpcG* results in a defect in PBS state transitions (Kondo et al. 2009). These data suggest that the deletion of *cpcG* reduces PBS function. In addition, Δ*cpcG* mutants also had a higher level of carotenoids than the WT (Oh and Montgomery 2019), similar to our observations (Supplementary Fig. S8). This has been linked to less sensitivity to oxidative stress (Oh and Montgomery 2019). It is therefore possible that the challenges that cells face at very high CO_2_ levels are related to reactive oxygen species (ROS). However, in our setup, the cells seem not to be significantly challenged by oxidative stress, because CRISPRi repression of several genes required for counter oxidative stress (*katG*/sll1987/, *sodB*/slr1516/, *tpx*/sll0755, *perR*/slr1738, *prxII*/sll1621) did not have much effect on growth under 30% CO_2_, or at least not more than under 4% CO_2_. Yet, this result is not conclusive, since the library works with single mutations, and the antioxidant genes mentioned here may have some functional redundancy. We tested the growth rate of a Δ*cpcG* mutant in optimal (4% CO_2_) and inhibitory high (30% CO_2_) conditions, concluding that the high levels of CO_2_ did not inhibit growth in the mutant. Even though this mutant’s growth was not significantly higher than that of the WT in high CO_2_, it is important to take into account that the light conditions considered optimal for the WT might differ in the case of a mutant with a defect in the light-harvesting structures. Kirst et al. (2014) showed that a mutant of *Synechocystis* with deletion of the *cpc* operon grows as well as the WT in 3% CO_2_ under light intensities of 800 μmol photons m^−2^ s^−1^ and greater. Altogether, this demonstrated that reducing the PBS light harvesting was beneficial when growing cultures with inhibitory high CO_2_ levels.

Another gene that might be related to photoinhibition is *pmgA*. Interestingly, the lowest fitness score observed in 30% CO_2_ was obtained with this gene. A study from Hihara et al. (1998) showed that *pmgA* is linked to the photosystem stoichiometry and acclimatization to high light levels. In that study, mutants with deletion of *pmgA* grow better under high light conditions initially, but they have a higher sensitivity to light stress in the long term. Results from Miao et al. (2023) showed that when comparing low light and low carbon cultures of *Synechocystis* with and without *N*-(3,4-dichlorophenyl)-*N*-dimethylurea (DCMU), which inhibits the activity of PSII by blocking the electron transfer from PSII to plastoquinone, there is no advantage in suppressing *pmgA*. This agrees with the idea that *pmgA* repression might increase photosynthetic activity since under conditions where PSII is already inactivated, the repression of *pmgA* does not have an effect. Using the database from that study (Miao et al. 2023), it can also be seen that the knockdown of *pmgA* is beneficial under 1% CO_2_, regardless of the light intensity. These results do not completely align with those of Hihara et al. (1998). However, it is essential to remember that CRIPRi libraries are knockdowns, and the results of Hihara et al. (1998) came from a deletion strain. It could be that a reduced expression of *pmgA* is not detrimental in high light conditions and 1% CO_2_, while deletion is. In our study, the high effect of the knockdown of *pmgA* in 30% CO_2_ could be linked with both the lack of regulation of the photosystems for the acclimatization to high light levels and the deficient regulation of carbon utilization pathways.

We also considered that the reason CRISPRi repression of PC and APC synthesis was beneficial for growth under 30% CO_2_ could be due to a smaller nitrogen requirement by cells with less PBS. Assimilation of nitrate into proteins is energetically expensive and the cells may grow faster if they do not have to make a lot of PBS. This is because PBS may account for up to 60% of the protein mass in cyanobacteria (Singh et al. 2015) although the content of PBS is highly flexible depending on the needs of the cell. For example, under nitrogen starvation, the cellular contents of PBS and nitrogen drop dramatically (Görl et al. 1998; Collier and Grossman 1992, 1994; Möllers et al. 2014; Singh et al. 2015). However, our Δ*cpcG* mutant did not have a significantly reduced content of PBS (Fig. 7b) and did not show a different growth rate in 30% CO_2_ and 4% CO_2_ (Fig. 7c). This led us to the conclusion that a reduction of PBS in the Δ*cpcG* cells was not beneficial in high CO_2_ because of the benefit of a higher C/N ratio in the biomass, but because of the reduction of light harvesting by the PBS. A different possibility suggested by previous studies is that the allocation of cellular protein is suboptimal in *Synechocystis* under certain growth conditions (Jahn et al. 2018; Miao et al. 2023) and that this may be the case for 30% CO_2_ as well. Therefore, knockdown of the PBS antenna obtained by CRISPRi repression of *cpc* and *apc* genes could be beneficial under conditions where the antenna is not needed (Miao et al. 2023). Given that light harvesting to obtain energy for CO_2_ concentration is not necessary in our cultures with 30% CO_2_, it is possible that the biosynthetic capacity of the cells was spent in a manner that was more beneficial to the cell than PBS biosynthesis.

We still do not fully understand why *Synechocystis* grew much slower under 30% CO_2_ than under optimal CO_2_ conditions at intermediate light intensities. While the downregulation of certain pathways, such as light harvesting and electron transfer, alleviated some of the growth inhibition, the underlying cause remains elusive. It is noteworthy that 30% CO_2_ did not inhibit growth under low light levels, where growth was light-limited and the growth rate significantly lower than under optimal conditions (Fig. 6). If the light intensity is increased to levels that are optimal for growth under 4% CO_2_, a condition of “metabolic congestion” may occur under 30% CO_2_ downstream of the photochemical and CO_2_ fixation processes, which could explain the growth inhibition. This could be due to metabolic imbalances caused by high intracellular CO_2_ concentrations, such as the accumulation of inhibitory metabolites due to excessive CO_2_ fixation or the exhaustion of essential metabolites that prevents optimal metabolic fluxes. Future research should aim to identify the precise causes of this congestion and develop strategies to alleviate them.

## Supplementary Information

Below is the link to the electronic supplementary material.

Supplementary file1 (PDF 5680 KB)

Supplementary file2 (XLSX 930 KB)

## Data Availability

Raw sequencing data were deposited at the European Nucleotide Archive (ENA accession number PRJEB79744). Source code used for analyses is available on GitHub (https://github.com/ute-hoffmann/CO2_cyano_CRISPRi) and Zenodo (10.5281/zenodo.14095552).
