# Supplementary material for: Photosynthesis in Synechocystis sp. PCC 6803 is not optimally regulated under very high CO2
Source: Appl Microbiol Biotechnol. 2025 Jan 30;109(1):33. doi: 10.1007/s00253-025-13416-2 (PMC11782454; doi:10.1007/s00253-025-13416-2)
Supplement: Supplementary file 1 — Supplementary file1 (PDF 5680 KB) [file 253_2025_13416_MOESM1_ESM.pdf]

Supplementary figures

Applied Microbiology and Biotechnology

Photosynthesis in *Synechocystis* sp. PCC 6803 is not optimally regulated under very high CO_2

Elena Carrasquer-Alvarez[1], Ute Angelika Hoffmann[2], Adrian Sven Geissler[3], Axel Knave[2], Jan Gorodkin[3], Stefan Ernst Seemann[3], Elton Paul Hudson[2], Niels-Ulrik Frigaard[1]*

[1] Marine Biological Section, Department of Biology, University of Copenhagen, Helsingør, Denmark

[2] School of Engineering Sciences in Chemistry, Biotechnology and Health, Science for Life Laboratory, KTH—Royal Institute of Technology, Stockholm, Sweden

[3] Center for non-coding RNA in Technology and Health, Department of Veterinary and Animal Sciences, University of Copenhagen, Frederiksberg, Denmark

* Corresponding author. E-mail: nuf@bio.ku.dk

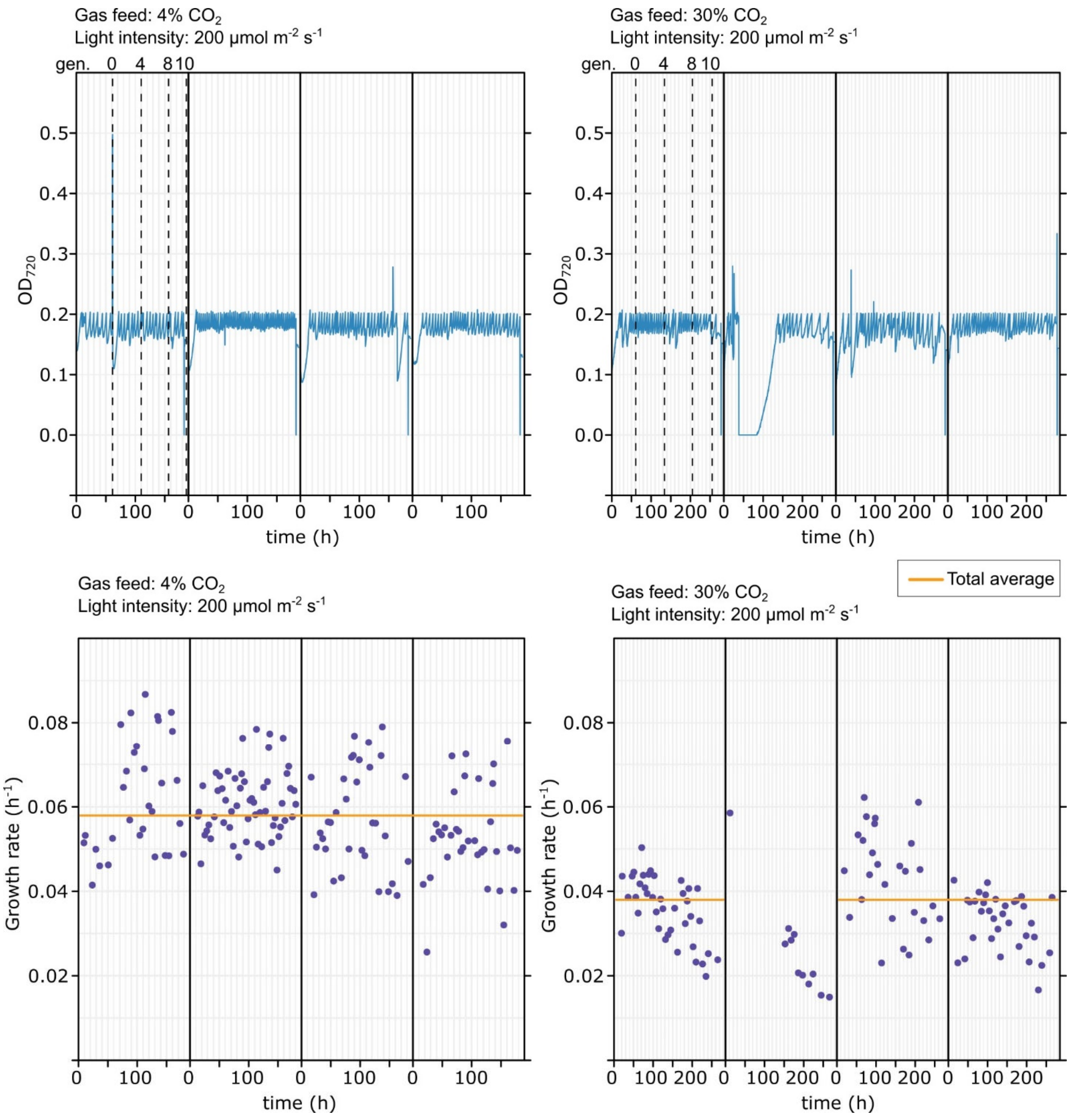

Suppl. Fig. S1 Turbidostatic cultivations of the *Synechocystis* CRISPRi mutant library under 4% and 30% CO_2

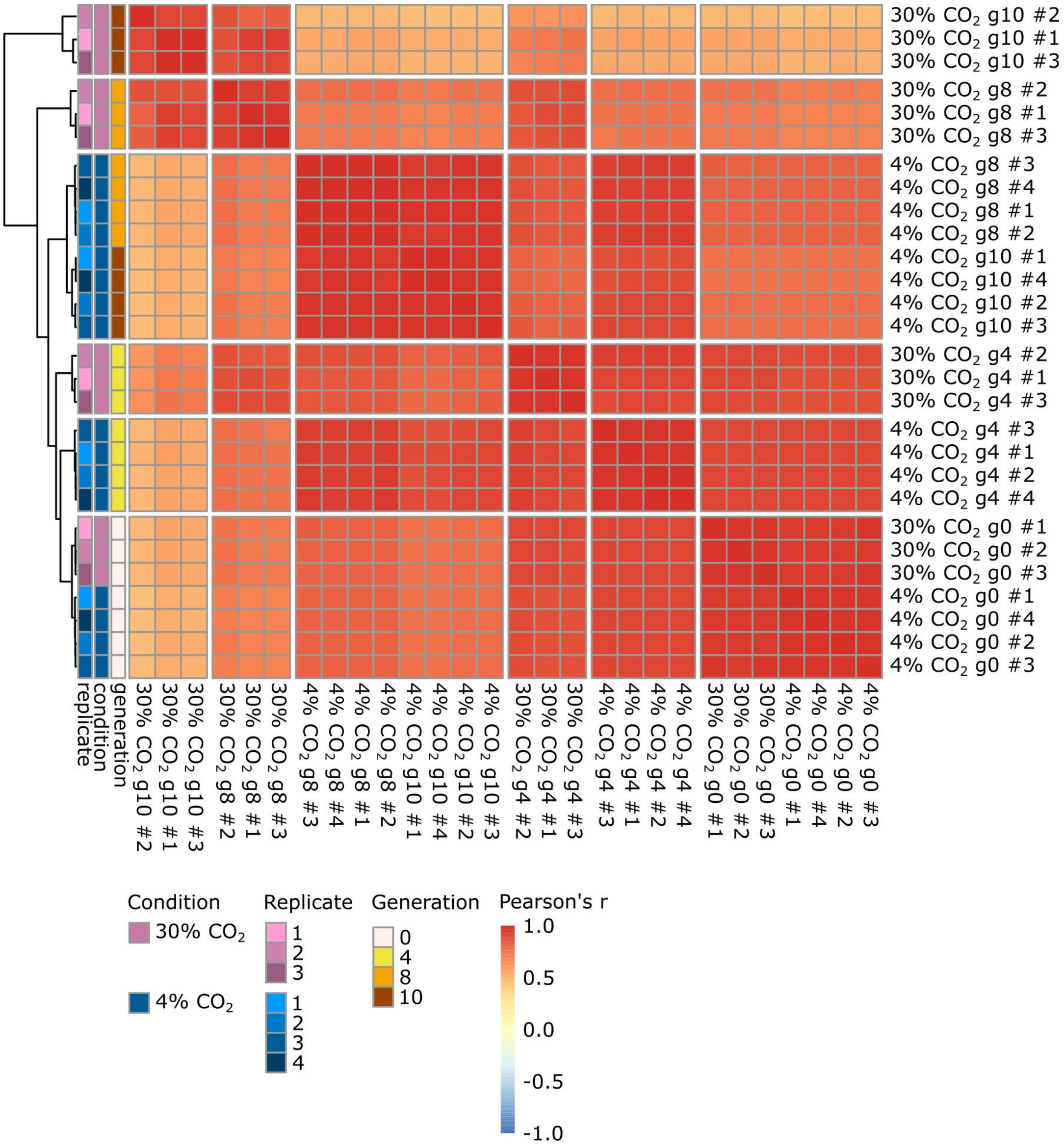

Suppl. Fig. S2 Replicate analysis of the turbidostatic cultivations of the *Synechocystis* CRISPRi mutant library

a

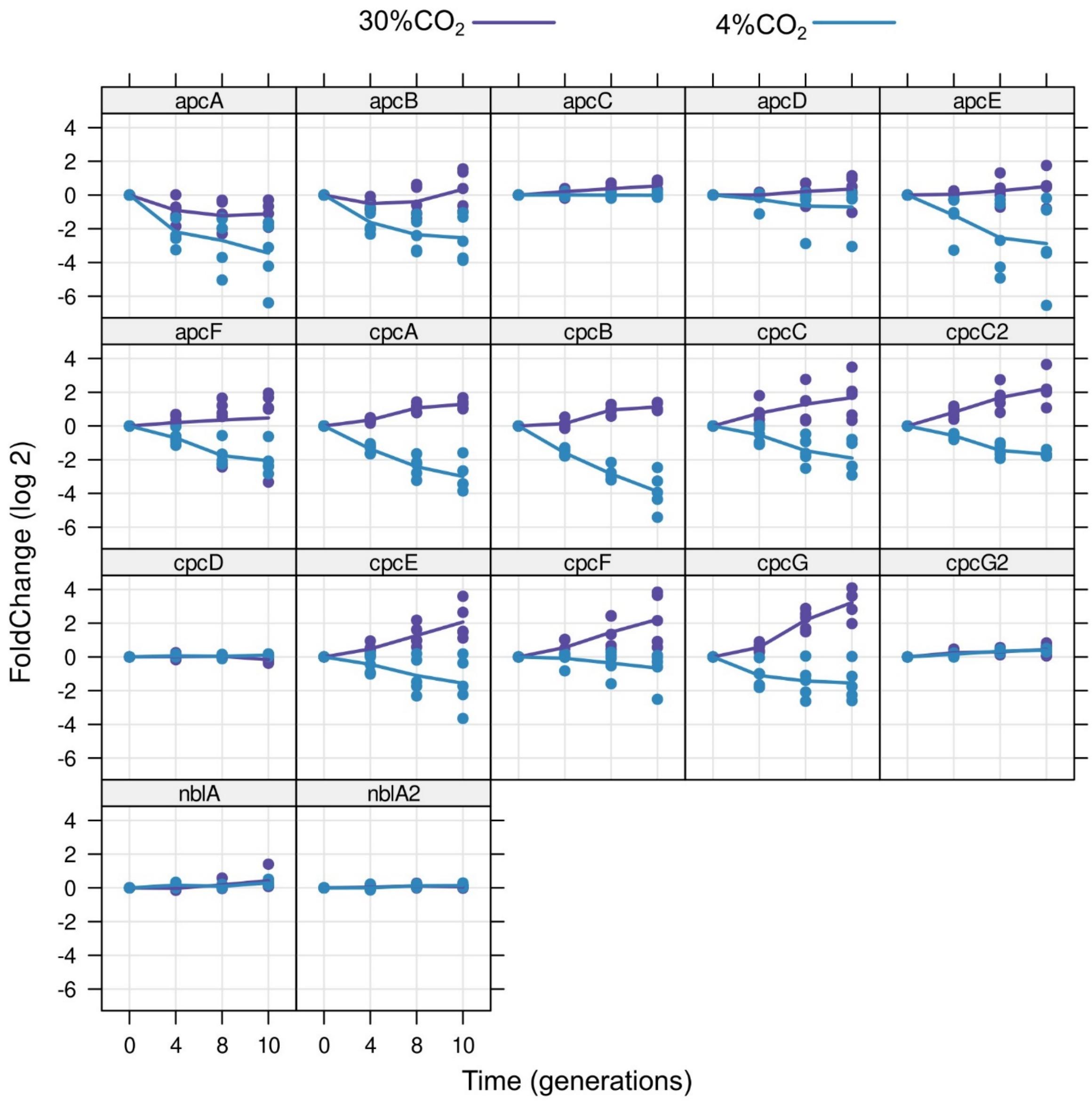

Suppl. Fig. S3 Change in abundance of selected *Synechocystis* CRISPRi mutants during turbidostatic cultivations. **(a)** Genes related to phycobilisomes. **(b)** Genes related to photosystems I and II. **(c)** Miscellaneous genes.

b

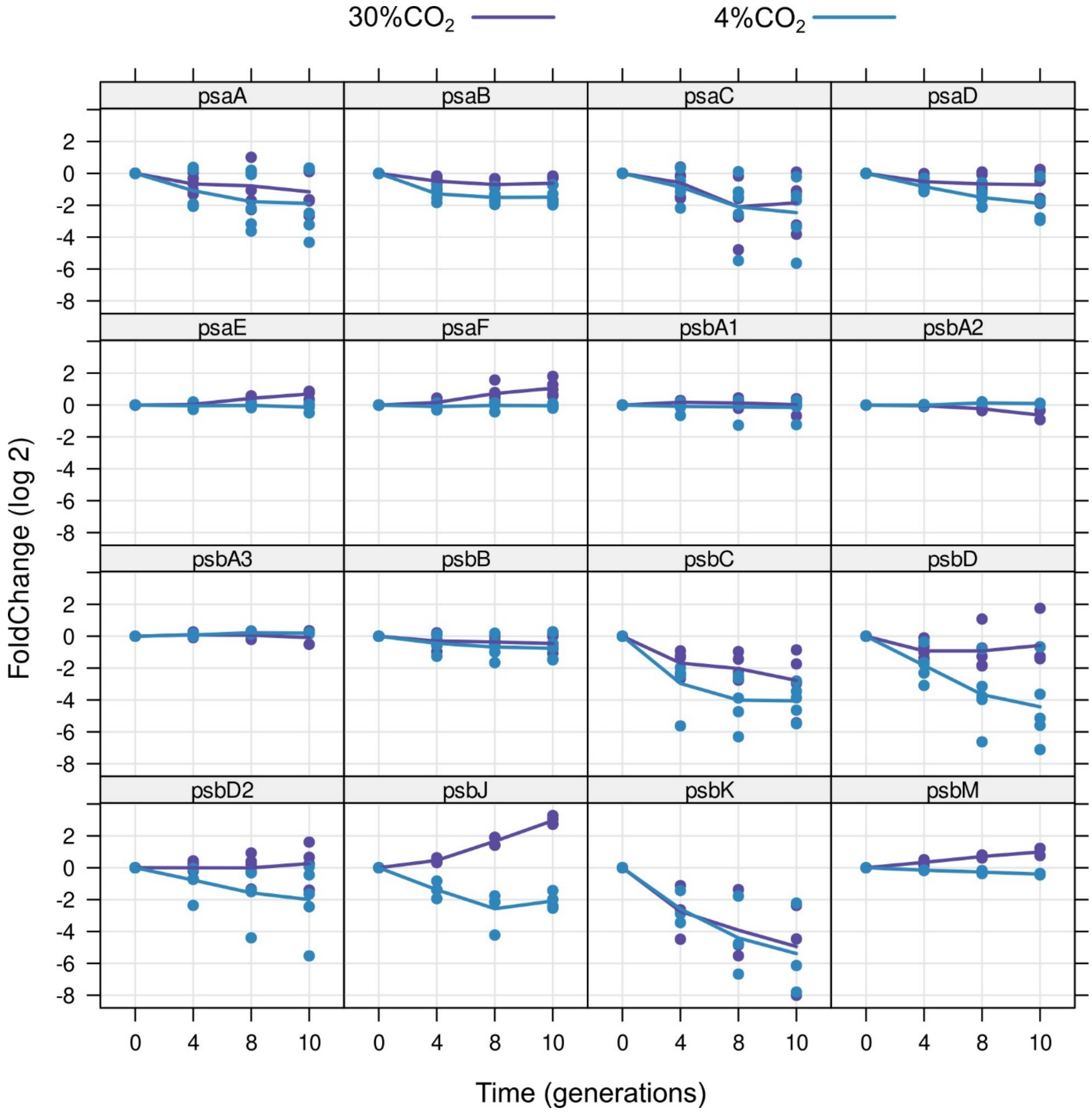

Suppl. Fig. S3 Change in abundance of selected *Synechocystis* CRISPRi mutants during turbidostatic cultivations. **(a)** Genes related to phycobilisomes. **(b)** Genes related to photosystems I and II. **(c)** Miscellaneous genes.

c

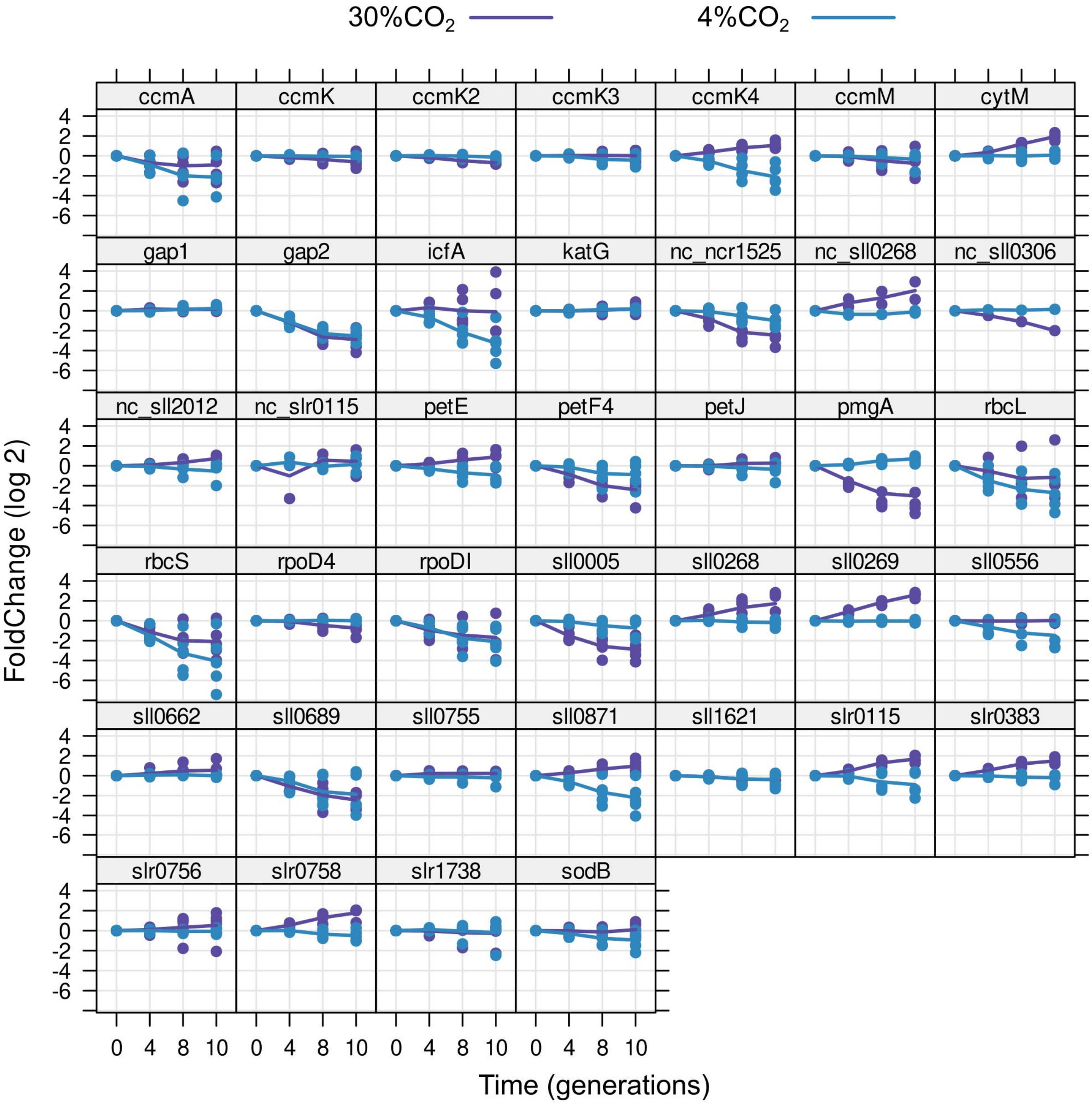

Suppl. Fig. S3 Change in abundance of selected *Synechocystis* CRISPRi mutants during turbidostatic cultivations. **(a)** Genes related to phycobilisomes. **(b)** Genes related to photosystems I and II. **(c)** Miscellaneous genes.

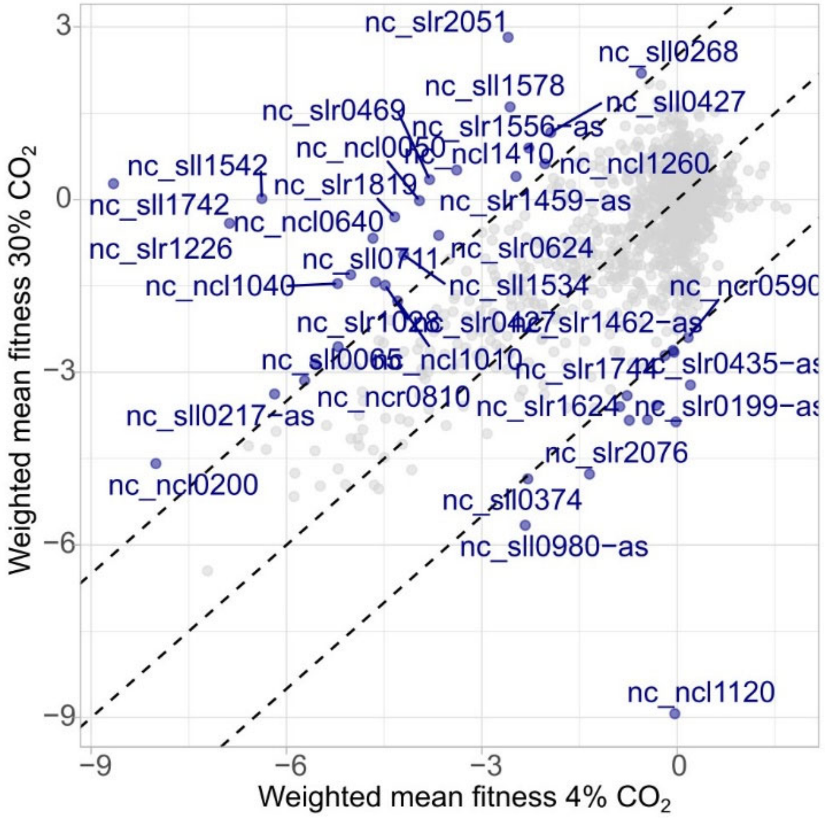

Suppl. Fig. S4 Dot plot of fitness scores at 30% CO_2 versus fitness scores at 4% CO_2 of CRISPRi knockdown mutations of non-coding targets

a

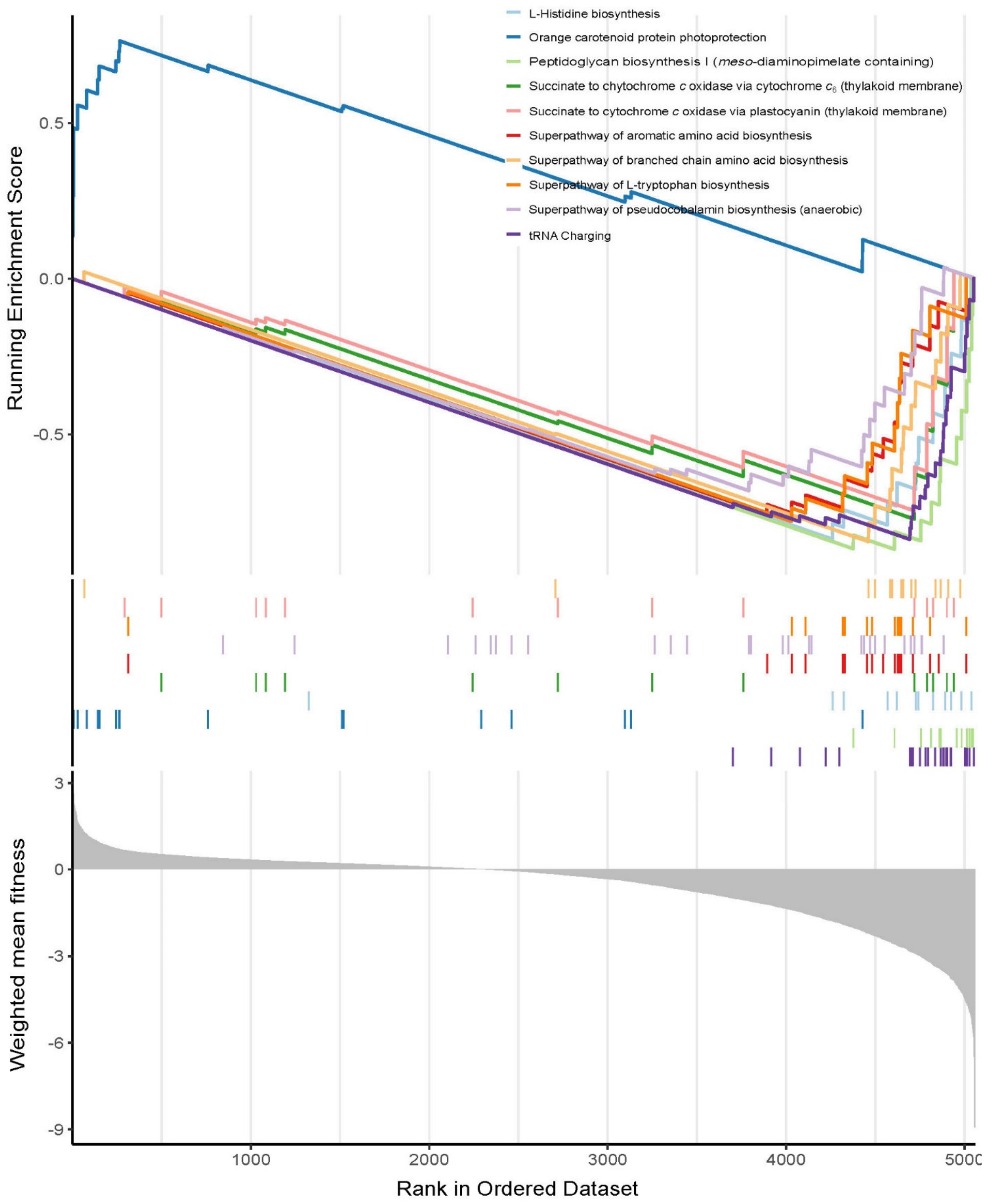

Suppl. Fig. S5 GSEA plots of selected pathways. **(a)** Pathways from CyanoCyc (https://cyanocyc.org/). **(b)** Pathways from GO knowledgebase (https://geneontology.org/). **(c)** Pathways from KEGG (https://www.kegg.jp/)

b

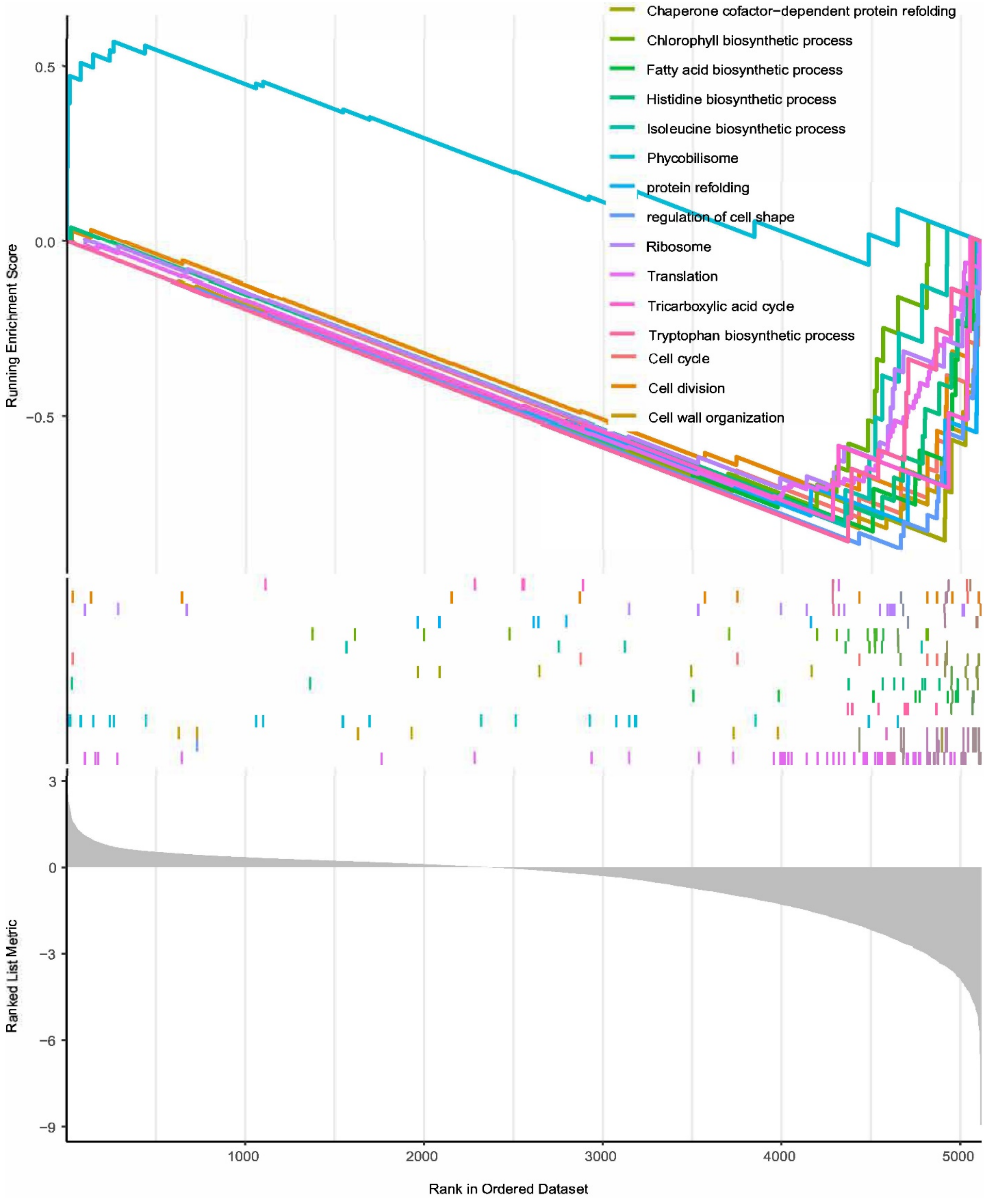

Suppl. Fig. S5 GSEA plots of selected pathways. **(a)** Pathways from CyanoCyc (https://cyanocyc.org/). **(b)** Pathways from GO knowledgebase (https://geneontology.org/). **(c)** Pathways from KEGG (https://www.kegg.jp/)

c

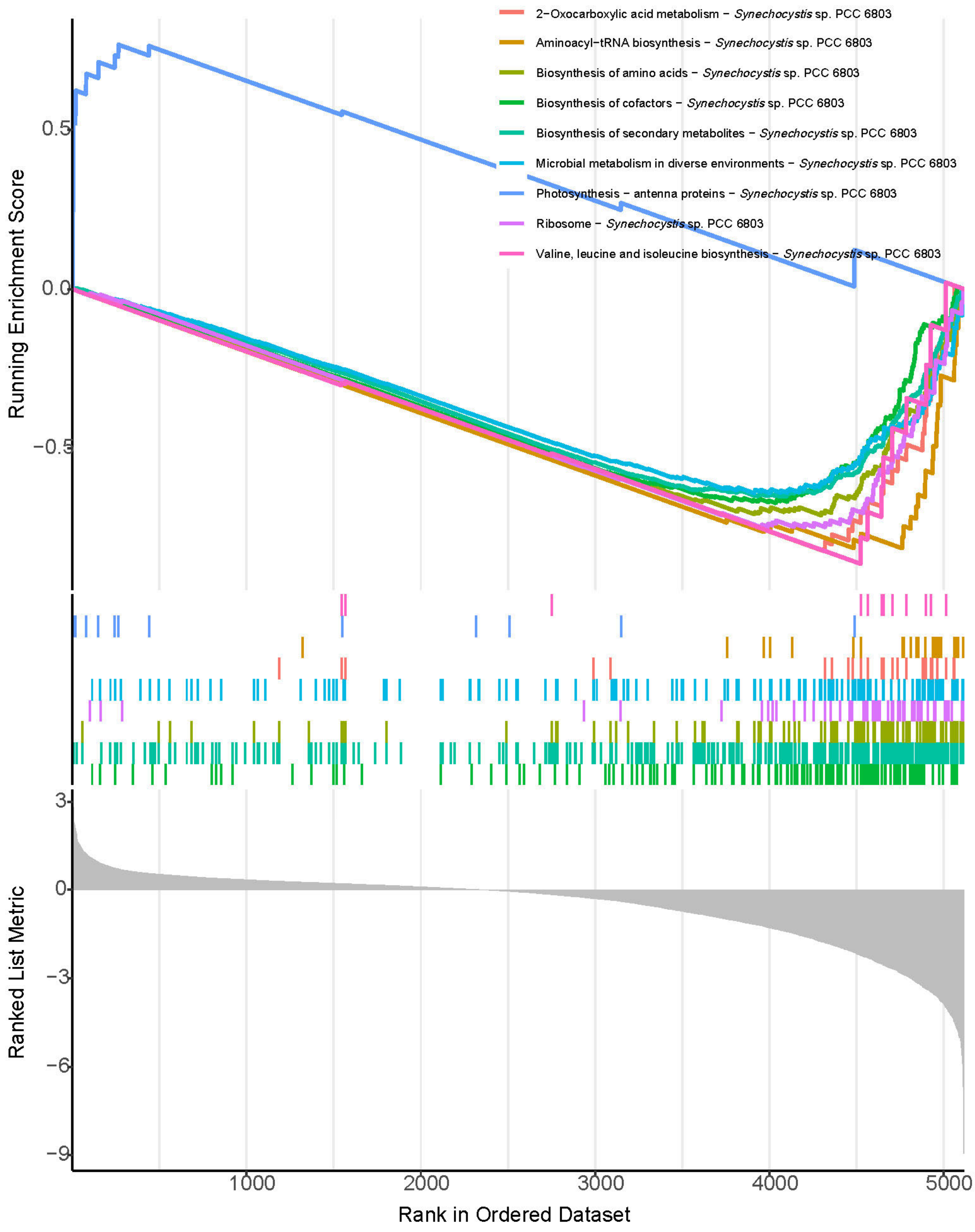

Suppl. Fig. S5 GSEA plots of selected pathways. **(a)** Pathways from CyanoCyc (https://cyanocyc.org/). **(b)** Pathways from GO knowledgebase (https://geneontology.org/). **(c)** Pathways from KEGG (https://www.kegg.jp/)

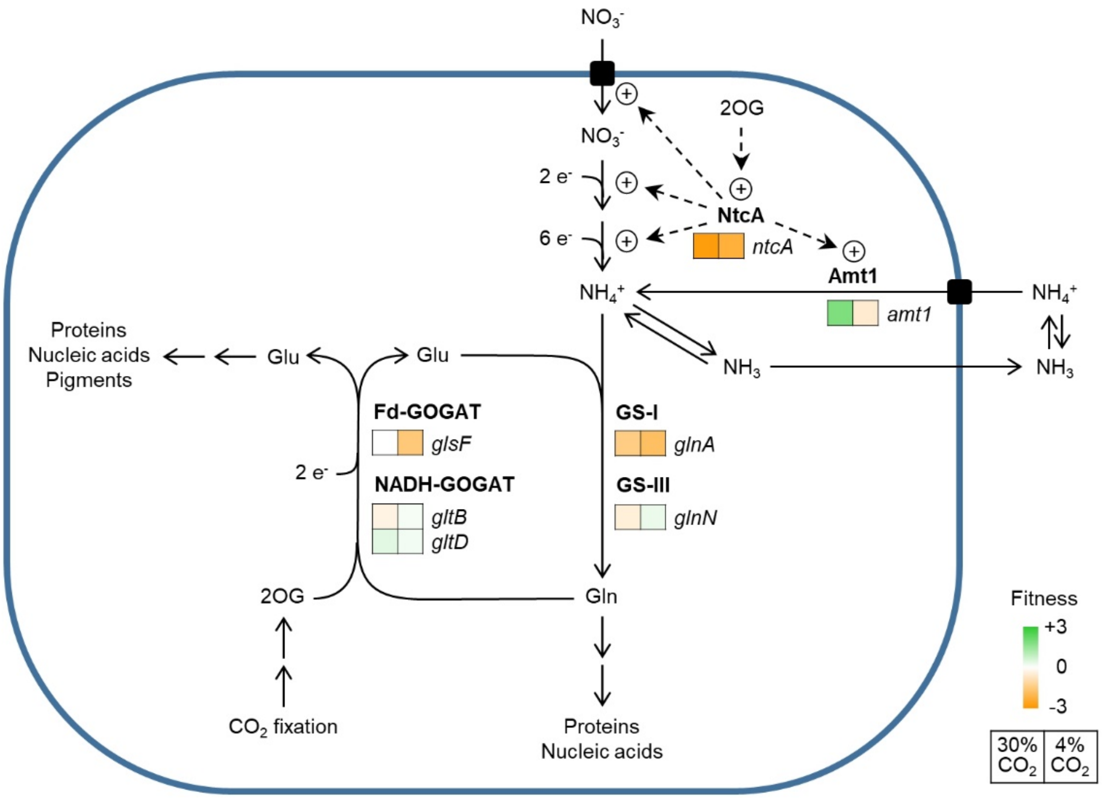

Suppl. Fig. S6 Simplified pathways in *Synechocystis* of nitrate and ammonium assimilation (derived from Ohashi et al. 2011 and Esteves-Ferreira et al. 2018). Heatmap-visualized fitness scores of the CRISPRi screen of genes in 30% and 4% CO_2 are shown (from Supplementary Table S1). Other genes than those shown are involved in nitrogen metabolism; the CRISPRi repression data of some of these were inconclusive. Abbreviations: 2OG, 2-oxo-glutarate; Gln, glutamine; Glu, glutamate; GOGAT, glutamine:2-oxo-glutarate aminotransferase; GS, glutamine synthetase.

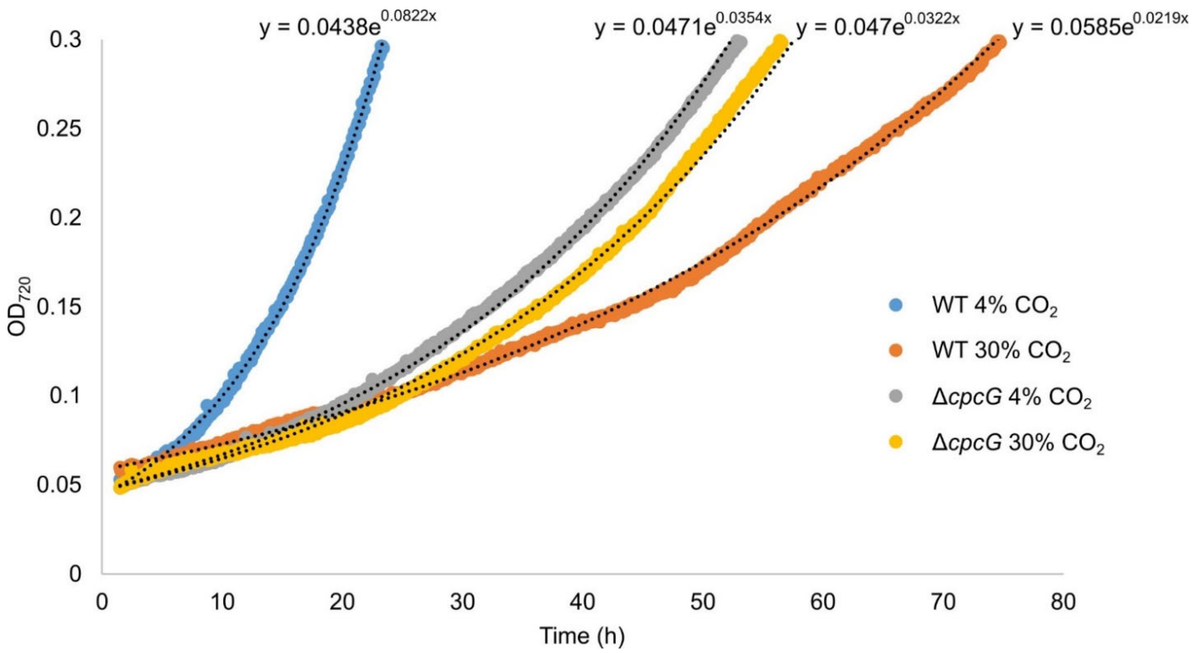

Suppl. Fig. S7 Growth curves of WT and Δ*cpcG* mutant in batch culture with 4% and 30% CO_2

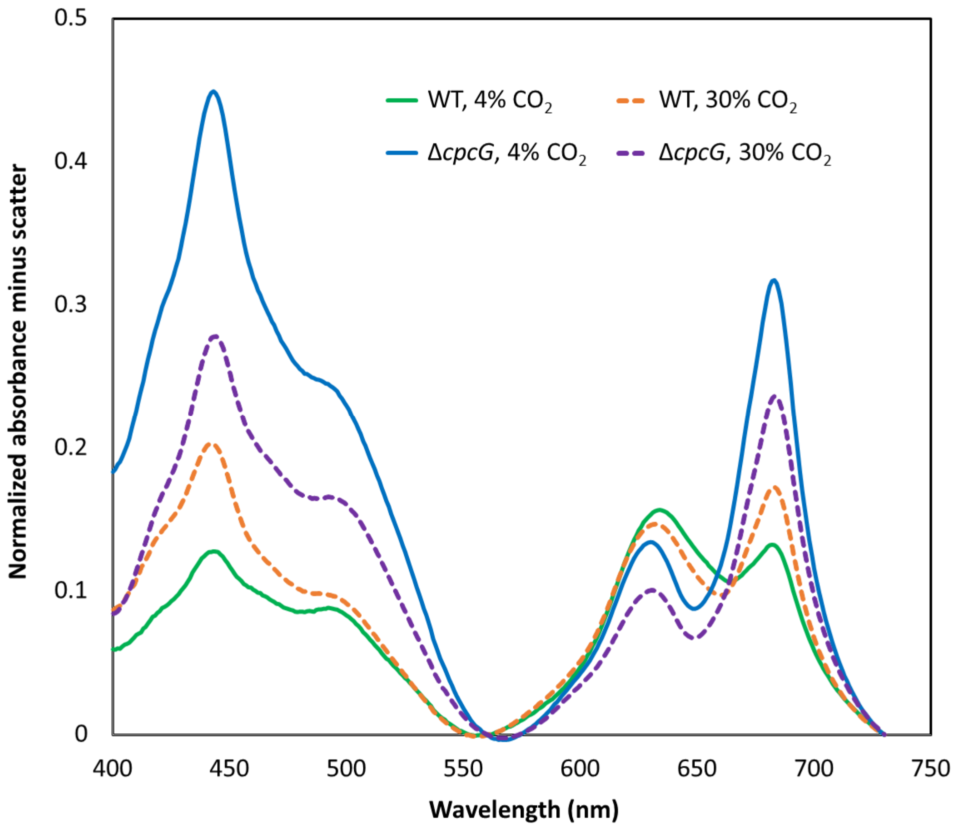

Suppl. Fig. S8 Normalized absorption spectra of WT and Δ*cpcG* mutant from liquid cultures with either 4% or 30% CO_2. The samples and absorption spectra normalization are identical to those described in Fig. 7b. The absorption peaks around 500 nm are mostly due to carotenoids because chlorophyll *a* and the phycobiliproteins have negligible absorption here (Fuente et al. 2021).

References

Esteves-Ferreira AA, Inaba M, Fort A, Araújo WL, Sulpice R (2018) Nitrogen metabolism in cyanobacteria: metabolic and molecular control, growth consequences and biotechnological applications. Critic Rev Microbiol 44(5):541–560. doi: 10.1080/1040841X.2018.1446902

Fuente D, Lazar D, Oliver-Villanueva JV, Urchueguía JF (2021) Reconstruction of the absorption spectrum of *Synechocystis* sp. PCC 6803 optical mutants from the in vivo signature of individual pigments. Photosynth Res 147:75–90. doi: 10.1007/s11120-020-00799-8

Ohashi Y, Shi W, Takatani N, Aichi M, Maeda S, Watanabe S, Yoshikawa H, Omata T (2011) Regulation of nitrate assimilation in cyanobacteria. J Exp Bot 62(4):1411–1424. doi: 10.1093/jxb/erq427
